# Quantile regression for static panel data models with time-invariant regressors

**DOI:** 10.1371/journal.pone.0289474

**Published:** 2023-08-02

**Authors:** Li Tao, Lingnan Tai, Maozai Tian

**Affiliations:** 1 School of Information, Beijing Wuzi University, Beijing, China; 2 Center for Applied Statistics, School of Statistics, Renmin University of China, Beijing, China; 3 School of Statistics and Information, Xinjiang University of Finance and Economics, Xinjiang, China; Semnan University, IRAN, ISLAMIC REPUBLIC OF

## Abstract

This paper proposes two new weighted quantile regression estimators for static panel data model with time-invariant regressors. The two new estimators can improve the estimation of the coefficients with time-invariant regressors, which are computationally convenient and simple to implement. Also, the paper shows consistency and asymptotic normality of the two proposed estimator for sequential and simultaneous *N*, *T* asymptotics. Monte Carlo simulation in various parameters sets proves the validity of the proposed approach. It has an empirical application to study the effects of the influence factors of China’s exports using the trade gravity model.

## Introduction

Panel data have their own distinct data characteristics, compared with simple cross-sectional data and time series data. Panel data have more observations, which can improve the validity of econometric model estimation. Besides, panel data can control the effect of omitted variables when modeling and improve the estimation accuracy of the estimation. The traditional panel data analysis is mainly based on the conditional mean regression methods, which cannot fully describe the data. Quantile regression for panel date can describe the independent variable for the dependent variable range accurately, and capture systematic influences of covariates on the location, scale and shape of the conditional distribution of the response.

Recently, there has been a growing literature on studying quantile regression for static panel data. Koenker [1] proposed quantile regression with fixed effects (FEQR) and penalized quantile regression with fixed effect (PQR) employing *l*_1_ regularization methods, pointed out that shrinkage of a large number of individual fixed effects toward a common value in the panel model can help to modify the variability caused by these individual effects. Lamarche [2] proved that there existing optimal penalty parameter of penalized quantile regression for panel data with fixed effect. However, when the sample size is large, the calculation of the penalty estimation is rather complicate. Moreover, when the panel data model contains time-invariant variables, the penalty estimator is less effective in estimating the time-invariant variables.

Canay [3] introduceed a two-step estimator for panel data quantile regression models. The two-step estimation method eliminates the fixed effect in the first step, which can greatly reduce the estimated parameters in quantile regression and avoid the choice of the penalty parameters. Obviously, when there are non time-varying variables in the panel model, the two-stage estimation method will ignore the estimation of the coefficient of the non time-varying covariates.

Galvao and Wang [4] developed a new minimum distance quantile regression (MD-QR) estimator for panel data models with fixed effects, which is computationally fast, especially for large cross-sections. Galvao et al. [5] proved that unbiased asymptotic normality of both the FEQR and MD-QR estimators, showing that quantile regression is applicable to the same type of panel data (in terms of *N*, *T*) as other nonlinear models. However, the MD-QR estimator is not applicable to the case that the panel model contains the time-invariant independent variables, which is defined as the weighted average of the individual quantile regression slope estimators. There have been other growing studies on quantile regression for panel data. See, for example, Harding and Lamarche [6, 7], Galvao et al. [8], Tao et al. [9], Dai et al. [10], Dai and Jin [11]. The existing research on panel data models mainly focuses on obtaining estimates of time-varying covariates. However, when the panel model contains time-varying covariates, most of these methods are ineffective, or the estimation results are poor.

Some researchers have studied parameter estimation for panel models with time-invariant covariates based on mean regression methods. Plümper and Troeger [12] suggested a three-stage procedure for the estimation of time-invariant in panel data models with unit effects. Pesaran and Zhou [13] proposed the fixed effects filtered (FEF) and fixed effects filtered instrumental variable (FEF-IV) estimators for estimation and inference in the case of time-invariant effects in static panel data models when *N* is large and *T* is fixed. Kripfganz and Schwarz [14] proposed a two-stage estimation procedure to identify the effects of time-invariant regressors in a dynamic version of the Hausman-Taylor model. Zhang and Zhou [15] used generalized method of moments (GMM) to estimate the time-varying effects in the first step, and run cross-sectional OLS regression of the time series average of the residuals to estimate the time-invariant effects in the second step.

To the best of our knowledge, our paper is the first to study quantile regression for panel data models with time-invariant regressors. We propose two weighted estimators of quantile regression. The minimum distance estimation and the two-step estimation of quantile regression fail, when panel date model exists time-invariant regressors. Therefore, we give two new estimators to improve the estimation of coefficients of time-invariant variables. First, considering the time-invariant regressor is exogenous, we propose a weighted estimator of quantile regression (W-QR). And then, regarding the time-invariant regressor is endogenous, we propose a weighted estimator of instrumental variable quantile regression (W-IVQR). The two new proposed methods need only two steps, which is computationally convenient and simple to implement. Regress dependent variables on time-varying variables with an intercept using the conventional quantile regression to obtain the slope and intercept estimators for each individual in the first step, and then use different weighted definitions to the obtained slope and intercept estimators to get the estimator of ***β*** and ***γ*** respectively in the second step. Besides, we study the asymptotic properties of W-QR and W-IVQR estimator under both sequential and simultaneous limits. Monte Carlo simulation in various parameters sets prove the validity of the proposed approach. Finally, we illustrate the proposed W-QR estimation with an application to analyze the effects of the influence factors of China’s exports using the trade gravity model.

The rest of the paper is organized as follows. Section 2 gives the static panel data model with time-invariant regressors and proposes the W-QR estimator and the W-IVQR estimator. Section 3 is devoted to the asymptotic behavior of the proposed estimators. Section 4 describes the Monte Carlo simulation. In Section 5, we illustrate the new approaches with an application to analyze the effects of the influence factors of China’s exports using the trade gravity model. In the end, Section 6 summarizes the paper.

## The model and estimators

### Static panel data model with time-invariant regressors

Consider the panel data model that contains time-varying as well as time-invariant regressors:

$$\begin{array}{c} y_{it}=\eta_{i}+{\text{z}}_{i}^{\prime }\gamma +{\text{x}}_{it}^{\prime }\beta +\varepsilon_{it},i=1,2,\ldots ,N;t=1,2,\ldots ,T, \end{array}$$ (1)

where **x**_*it*_ is a *q* × 1 vector of time-varying variables, and **z**_*i*_ is a *p* × 1 vector of observed individual-specific variables that only vary over the cross-sectional unit *i*. In addition to **z**_*i*_, the outcomes, *y*_*it*_, are also governed by unobserved individual specific effects, *η*_*i*_. The focus of the analysis is on estimation and inference involving the elements of ***β*** and ***γ***. It is clear that without further restrictions on *η*_*i*_, ***γ*** cannot be identified even if ***β*** is known to the researchers. For example, consider the simple case where ***β*** = 0, and assume that *T* is small. Then averaging across *t*, we obtain

$${\bar{y}}_{i}={\text{z}}_{i}^{\prime }\gamma +v_{i},$$

where ${\bar{y}}_{i}=T^{-1}\Sigma_{t=1}^{T}y_{it}$, $v_{i}=\eta_{i}+{\bar{\varepsilon }}_{i}$, and ${\bar{\varepsilon }}_{i}=T^{-1}\Sigma_{t=1}^{T}\varepsilon_{it}$. It is clear that without specifying how *v*_*i*_ and **z**_*i*_ are related it will not be possible to identify the **z**_*i*_. To deal with this problem, it is often assumed that there exists instruments that are uncorrelated with *v*_*i*_, but at the same time are sufficiently correlated with **z**_*i*_. Even if such instruments exist, a number of further complications arise if ***β*** ≠ 0. In such a case, the instrumental variable approach must be extended also to deal with the possible dependencies between *η*_*i*_ and **x**_*it*_. In what follows we allow for **x**_*it*_ and *η*_*i*_ to have any degree of dependence, but initially assume that **z**_*i*_ and *v*_*i*_ are uncorrelated for identification of ***γ***.

It is convenient to write model (1) in matrix form as,

$$\begin{array}{c} \text{y}=\text{D}\eta +\text{Z}\gamma +\text{X}\beta +\varepsilon , \end{array}$$ (2)

where **y** = (*y*_*it*_) is a *NT* × 1 matrix, $\text{z}={\left({\text{z}}_{1}^{\prime },\ldots ,{\text{z}}_{N}\right)}^{\prime }$, **Z** = **z** ⊗ **l**_*T*_ is a *NT* × *p* matrix, ⊗ is the Kronecker product, **X** = (**x**_*it*_) is a *NT* × *q* matrix, **D** = **I**_*N*_ ⊗ **l**_*T*_, **l**_*T*_ is a *T* × 1 vector of ones, ***η*** = (*η*_1_, ⋯, *η*_*N*_)′ is the *N* × 1 vector of individual specific effects or intercepts, and ***ε*** = (*ε*_*it*_) is a *NT* × 1 matrix. Note that **D** and **Z** represent an incidence matrix that identifies the *N* distinct individuals in the sample.

We assume that the *τ*th quantile of the error *ε*_*it*_(*τ*) is equal to zero. We consider the following model for the *τ*th conditional quantile functions of the response of the *t*th observation on the *i*th individual *y*_*it*_,

$$\begin{array}{c} Q_{y_{it}}\left(\tau |{\text{x}}_{it},{\text{z}}_{i},\eta_{i}\right)=\eta_{i}\left(\tau \right)+{\text{z}}_{i}^{\prime }\gamma \left(\tau \right)+{\text{x}}_{it}^{\prime }\beta \left(\tau \right). \end{array}$$ (3)

Galvao and Wang [4] propose a simple to implement and efficient minimum distance quantile regression estimator for panels with fixed effects. They consider a minimum distance quantile regression (MD-QR) estimator, ${\hat{\beta }}_{MD}$, defined as follows

$${\hat{\beta }}_{MD}=(\sum_{i=1}^{N}{\text{V}}_{i}^{-1}{)}^{-1}\sum_{i=1}^{N}{\text{V}}_{i}^{-1}{\hat{\beta }}_{i},$$

where ${\hat{\beta }}_{i}$ is the slope coefficient estimator from each individual quantile regression problem using the time-series data, and **V**_*i*_ denotes the associated variance-covariance matrix of ***β***_*i*_ for each individual. As we can see, MD-IVQR estimator is defined as the weighted average of the conventional QR slope estimators, with weights given by the inverses of the corresponding individual variance-covariance matrices, thus, when the model contains time-invariant regressors, MD-QR estimator can not identify ***γ***(*τ*) and *η*_*i*_(*τ*).

Chetverikov et al. [16] are primarily interested in estimating ***γ***(*τ*). They point out that (3) can also be considered as a special case in form of

$$\begin{array}{c} Q_{y_{it}}\left(\tau |{\text{x}}_{it},{\text{z}}_{i},{\tilde{\beta }}_{i}\right)={\tilde{\text{x}}}_{it}^{\prime }{\tilde{\beta }}_{i}\left(\tau \right),\;\tau \in U, \end{array}$$ (4)

$$\begin{array}{c} {\tilde{\beta }}_{i,1}\left(\tau \right)={\text{z}}_{i}^{\prime }\gamma \left(\tau \right)+\eta_{i}\left(\tau \right),\;\tau \in U. \end{array}$$ (5)

Indeed, setting ${\tilde{\text{x}}}_{it}=\left(1,{\text{x}}_{it}\right)$, and assuming that ${\tilde{\beta }}_{i}\left(\tau \right)=\left({\tilde{\beta }}_{i,1}\left(\tau \right),\beta \left(\tau \right)\right)$. To estimate ***γ***(*τ*), they develop the grouped IV quantile regression estimator which consists of the following two stages. At the first stage, for each *τ* and *i*, estimate *τ*th quantile regression of *y*_*it*_ on ${\tilde{\text{x}}}_{it}$ by the classical quantile regression estimator of Koenker and Bassett [17]. Estimate a 2SLS regression of ${\hat{\tilde{\beta }}}_{i,1}\left(\tau \right)$ on **z**_*i*_ using the instrument to get an estimator of ***γ***(*τ*) at the second stage. The differences between our study and Chetverikov et al. [16] lie in three aspects: (1) Chetverikov et al. [16] consider a more general model that allows for interaction via ${\tilde{\beta }}_{i}\left(\tau \right)$, the panel data model (3) studied in this paper is a special case of (4)-(5) in form and has no interaction effect; (2) our study is interested in estimating ***β***(*τ*) and ***γ***(*τ*), Chetverikov et al. [16] are primarily interested in estimating ***γ***(*τ*) without considering the estimation of ***β***(*τ*); (3) there are different requirements for *N* and *T* tending to infinity in asymptotic theory, Chetverikov et al. [16] assume that $\frac{N^{2/3}\text{log}\;T}{T}\to 0$ as *N* → ∞, our study assumes that $\frac{N^{2}\left(\text{log}\;N\right)}{T}{\left|\text{log}\frac{{\left(\text{log}\;N\right)}^{0.5}}{T^{0.5}}\right|}^{2}\to 0$ as *N* → ∞.

### The W-QR estimator

In this section, we consider the case when the time-invariant regressor **z**_*i*_ is exogenous, that is, *E*(**z**_*i*_*η*_*i*_(*τ*)) = 0. The weighted estimator of quantile regression (W-QR) estimator can be computed as follows.

Step 1: For each individual *i* and each quantile index *τ* from the set *U* of indices of interest, estimate *τ*th quantile regression of *y*_*it*_ on the time-varying regressors **x**_*it*_ by the classical quantile regression estimator of Koenker and Bassett [17]:

$$\begin{array}{c} \left({\hat{\alpha }}_{i}\left(\tau \right),{\hat{\beta }}_{i}\left(\tau \right)\right)=\text{arg}\;\underset{\beta \in R^{q},\alpha_{i}\in R^{1}}{\text{min}}\;\sum_{t=1}^{T}\rho_{\tau }\left(y_{it}-\alpha_{i}\left(\tau \right)-{\text{x}}_{it}^{\prime }\beta \left(\tau \right)\right), \end{array}$$ (6)

where *ρ*_*τ*_(*u*) = *u*(*τ* − *I*(*u* < 0)) is the check function. We can see that ${\hat{\beta }}_{i}\left(\tau \right)$ and ${\hat{\alpha }}_{i}\left(\tau \right)$ are the slope and intercept estimator for each individual quantile regression problem with using the time-series data.

Step 2: Follow Galvao and Wang [4], and like Chetverikov et al. [16] and Pesaran and Zhou [13], the W-QR estimator is computed as

$$\begin{array}{c} {\hat{\gamma }}_{W-QR}\left(\tau \right)=[\sum_{i=1}^{N}({\text{z}}_{i}-\bar{\text{z}})({\text{z}}_{i}-\bar{\text{z}}{)}^{\prime }{]}^{-1}\sum_{i=1}^{N}({\text{z}}_{i}-\bar{\text{z}})(\hat{\alpha_{i}}\left(\tau \right)-\bar{\hat{\alpha }}\left(\tau \right)), \end{array}$$ (7)

$$\begin{array}{c} {\hat{\beta }}_{W-QR}\left(\tau \right)={\left(\sum_{i=1}^{N}{\text{V}}_{i}^{-1}\right)}^{-1}\sum_{i=1}^{N}{\text{V}}_{i}^{-1}{\hat{\beta }}_{i}\left(\tau \right), \end{array}$$ (8)

$$\begin{array}{c} {\hat{\eta }}_{i,W-QR}\left(\tau \right)={\bar{y}}_{i}-{\text{z}}_{i}^{\prime }{\hat{\gamma }}_{W-QR}\left(\tau \right)-{\bar{\text{x}}}_{i}^{\prime }{\hat{\beta }}_{W-QR}\left(\tau \right), \end{array}$$ (9)

where $\bar{\text{z}}=N^{-1}\sum_{i=1}^{N}{\text{z}}_{i}$, $\bar{\hat{\alpha }}\left(\tau \right)=N^{-1}\sum_{i=1}^{N}\hat{\alpha_{i}}\left(\tau \right)$, ${\bar{\text{x}}}_{i}=T^{-1}\sum_{t=1}^{T}{\text{x}}_{it}$ and **V**_*i*_ denotes the associated variance-covariance matrix of ${\hat{\beta }}_{i}$ for each individual. As we can see that the estimators ${\hat{\beta }}_{i}$ and ${\hat{\alpha }}_{i}$ gained in the first step are weighted in different forms separately to get the W-QR estimator of $\hat{\beta }$ and $\hat{\gamma }$ in the second step. According to the above formulas (7) and (8), the $\hat{\beta }$ is a weighted combination of ${\hat{\beta }}_{i}$, and $\hat{\gamma }$ is a weighted combination of centralized ${\hat{\alpha }}_{i}$. In addition, the definition of ${\hat{\eta }}_{i,W-QR}\left(\tau \right)$ makes sense because ${\hat{\eta }}_{i}\left(\tau \right)$ is the intercept for cross-sectional unit *i*.

Remark 1: In mean regression, one need to compute FE estimator of ***β***, and then use the FE residuals to obtain ***γ***, see, Plümper and Troeger [12], Pesaran and Zhou [13]. That is to say, the mean regression methods to settle the estimation of model (1) need at least two stages. FE estimator of ***β*** is computed at the first stage, and the estimator of time invariant effects is gained at the second stage. Compared with the mean regression methods of parameter estimation in model (1), the proposed method need only two steps, which is computationally convenient and simple to implement. Regress *y*_*it*_ on **x**_*it*_ with an intercept using the conventional quantile regression to obtain the slope and intercept estimators for each *i* in the first step, and then use different weighted definitions to the obtained slope and intercept estimators to get the W-QR estimator of ***β*** and ***γ*** respectively in the second step.

Remark 2: However, the estimator ${\hat{\beta }}_{W-QR}\left(\tau \right)$, defined in (8) is infeasible in applications unless **V**_*i*_ is known for every individual. Thus, it is suggest to use the corresponding consistent estimator ${\hat{\text{V}}}_{i}$ to replace the unknown **V**_*i*_, then ${\hat{\beta }}_{W-QR}\left(\tau \right)$ is given by

$$\begin{array}{c} {\hat{\beta }}_{W-QR}\left(\tau \right)={\left(\sum_{i=1}^{N}{\hat{\text{V}}}_{i}^{-1}\right)}^{-1}\sum_{i=1}^{N}{\hat{\text{V}}}_{i}^{-1}{\hat{\beta }}_{i}\left(\tau \right). \end{array}$$ (10)

The specific form of $\hat{\text{V}}$ depends on the assumption on the dependence across individuals, examples for such estimators will be provided in formula (12).

### The W-IVQR estimator

In this section, we discuss the case when the time-invariant regressor **z**_*i*_ is exogenous. That is, we consider that **z**_*i*_ is correlated with *η*_*j*_(*τ*) or *ε*_*j*_(*τ*). We propose an IV version of W-QR estimator (denoted as W-IVQR) that allows for endogeneity of the time-invariant regressors.

We now provide W-IVQR estimator that allows for possible endogeneity of the time-invariant regressors, assuming the existence of *s* × 1 vector of instruments **r**_*i*_ for time-invariant **z**_*i*_, where **r**_*i*_ is independent of *η*_*j*_(*τ*) and *ε*_*j*_(*τ*) for all *i* and *j* and *s* ≥ *p*. The W-IVQR estimator can be obtained as follows.

Step 1: For each individual *i* and each quantile index *τ* from the set *U* of indices of interest, estimate *τ*th quantile regression of *y*_*it*_ on the time-varying regressors **x**_*it*_ by the classical quantile regression estimator of Koenker and Bassett [17]:

$$\begin{array}{c} \left({\hat{\beta }}_{i}\left(\tau \right),{\hat{\alpha }}_{i}\left(\tau \right)\right)=\text{arg}\;\underset{\beta \in R^{q},\alpha_{i}\in R^{1}}{\text{min}}\;\sum_{t=1}^{T}\rho_{\tau }\left(y_{it}-{\text{x}}_{it}^{\prime }\beta \left(\tau \right)-\alpha_{i}\left(\tau \right)\right). \end{array}$$ (11)

We can see that ${\hat{\beta }}_{i}\left(\tau \right)$ and ${\hat{\alpha }}_{i}\left(\tau \right)$ are the slope and intercept estimator for each individual quantile regression problem with using the time-series data.

Step 2: Follow Galvao and Wang [4], and like Chetverikov et al. [16] and Pesaran and Zhou [13], the W-IVQR estimator is computed as

$${\hat{\gamma }}_{W-IVQR}\left(\tau \right)={\left({\text{Q}}_{\text{zr},N}{\text{Q}}_{\text{rr},N}^{-1}{\text{Q}}_{\text{zr},N}^{\prime }\right)}^{-1}\left({\text{Q}}_{\text{zr},N}{\text{Q}}_{\text{rr},N}^{-1}{\text{Q}}_{\text{r}\hat{\alpha }\left(\tau \right),N}\right),$$

$${\hat{\beta }}_{W-IVQR}\left(\tau \right)={\left(\sum_{i=1}^{N}V_{i}^{-1}\right)}^{-1}\sum_{i=1}^{N}V_{i}^{-1}{\hat{\beta }}_{i}\left(\tau \right),$$

and

$${\hat{\eta }}_{i,W-IVQR}\left(\tau \right)={\bar{y}}_{i}-{\text{z}}_{i}^{\prime }{\hat{\gamma }}_{W-IVQR}\left(\tau \right)-{\bar{\text{x}}}_{i}^{\prime }{\hat{\beta }}_{W-IVQR}\left(\tau \right),$$

where **Q**_**zr**,*N*_, **Q**_**rr**,*N*_ and ${\text{Q}}_{\text{r}\hat{\alpha }\left(\tau \right),N}$ are

$${\text{Q}}_{\text{zr},N}=N^{-1}\sum_{i=1}^{N}({\text{z}}_{i}-\bar{\text{z}})({\text{r}}_{i}-\bar{\text{r}}{)}^{\prime },\;{\hat{Q}}_{\text{rr}}=N^{-1}\sum_{i=1}^{N}({\text{r}}_{i}-\bar{\text{r}})({\text{r}}_{i}-\bar{\text{r}}{)}^{\prime },$$

$${\text{Q}}_{\text{r}\hat{\alpha }\left(\tau \right),N}=N^{-1}\sum_{i=1}^{N}({\text{r}}_{i}-\bar{\text{r}})(\hat{\alpha_{i}}\left(\tau \right)-\bar{\hat{\alpha }}\left(\tau \right)),$$

$\bar{\text{r}}=N^{-1}\sum_{i=1}^{N}{\text{r}}_{i}$, $\bar{\text{z}}=N^{-1}\sum_{i=1}^{N}{\text{z}}_{i},$ and $\bar{\hat{\alpha }}\left(\tau \right)=N^{-1}\sum_{i=1}^{N}\hat{\alpha_{i}}\left(\tau \right)$, ${\bar{y}}_{i}=T^{-1}\Sigma_{t=1}^{T}y_{it}$, ${\bar{\text{x}}}_{i}=T^{-1}\Sigma_{t=1}^{T}{\text{x}}_{it}$.

Remark 3: In Step 2, we estimate a centralized 2SLS regression of ${\hat{\alpha }}_{i}\left(\tau \right)$ on **z**_*i*_ using **r**_*i*_ as an instrument to get an estimator $\hat{\gamma }\left(\tau \right)$ of ***γ***(*τ*). The instrument **r**_*i*_ needs to satisfy the following two conditions: (i) instruments **r**_*i*_ can impact **z**_*i*_, and dim(**r**_*i*_) ≥ dim(**z**_*i*_); (ii) **r**_*i*_ is independent of *η*_*j*_(*τ*) and *ε*_*j*_(*τ*) for all *i* and *j*. In practice, for panel data model (1), we can choose Hausman and Taylor instrumental variables in [18]. Hausman and Talyor [18] proposed Hausman and Talyor (HT) estimator. One advantage of HT estimator is that there is no need for HT method to adapt instrumental variables beyond the model, but it requires the dimension of **x**_*it*_ which are uncorrelated with the individual effects is greater than **z**_*i*_ that are correlated with the individual effects. That is, we can use ${\bar{\text{x}}}_{i}$ as instrumental variables of **r**_*i*_, provided that *s* ≥ *p* in practice. HT instrumental variables have been widely used in quantile regression for panel data, e.g. see [6, 9].

## Asymptotic theory

Now we briefly discuss the asymptotic properties of the W-QR and W-IVQR estimators. We study the asymptotic properties of the two proposed estimators when both *T* and *N* go to infinity, both sequentially and simultaneously. The sequential asymptotics, denoted by (*T*, *N*)_*seq*_ → ∞, is defined as *T* diverging to infinity first, and then *N* → ∞. The simultaneous asymptotics, denoted by (*T*, *N*) → ∞, means *T* and *N* tend to infinity at the same time.

### Basic assumptions

To establish the asymptotic properties, we impose the following regularity conditions.

**Assumption 1**: (i) Observations are independent across individuals. (ii) For all *i* = 1, ⋯, *N*, (**x**_*it*_, *y*_*it*_) are i.i.d. within individuals.

**Assumption 2**: (i) For all *i* = 1, ⋯, *N* and *t* = 1, ⋯, *T*, the regressors, **x**_*it*_ satisfy the moment conditions ∥ **x**_*it*_ ∥ ≤ *C*_*M*_. (ii) For all *i* = 1, ⋯, *N*, all eigenvalues of $E[{\tilde{\text{x}}}_{it}{\tilde{\text{x}}}_{it}^{\prime }]$ are bounded from below by *c*_*M*_.

**Assumption 3**: For all *τ* ∈ *U*, (***β***(*τ*), ***γ***(*τ*), ***η***(*τ*)) is in the interior of the set B×G×T and B×G×T is compact. Put $u_{it}≔y_{it}-\eta_{i}-{\text{z}}_{i}^{\prime }\gamma -{\text{x}}_{it}^{\prime }\beta$, for each *δ* > 0,

$$\epsilon_{\delta }=\underset{1\leq i\leq N}{\text{inf}}\;\underset{|\eta_{i}\left|+\|\beta \|+\|\gamma \right\|=\delta }{\text{inf}}E\left[\int_{0}^{\eta_{i}+{\text{z}}_{i}^{\prime }\gamma +{\text{x}}_{it}^{\prime }\beta }\left\{F_{i}\left(s|{\text{z}}_{i},{\text{x}}_{it}\right)-\tau \right\}ds\right]>0,$$

where *F*_*i*_(⋅) is the distribution function of *u*_*it*_ conditional on **x**_*it*_ and **z**_*i*_.

**Assumption 4**: (i) Let *f*_*i*_(⋅) denote the conditional density function of *u*_*it*_ given **x**_*it*_ and **z**_*i*_, the conditional density function of *f*_*i*_(⋅) is continuously differentiable. For all *τ* ∈ *U*, *i* = 1, ⋯, *N*, *f*_*i*_(⋅) ≤ *C*_*f*_ and *f*_*i*_(0) ≥ *c*_*f*_. (ii) The derivative $f_{i}^{\prime }\left(\cdot \right)$ satisfying $|f_{i}^{\prime }\left(u_{it}|{\text{z}}_{i},{\text{x}}_{it}\right)|\leq C_{f}$.

Although in practice the observations are dependent across time for panel data, Assumption 1 is usual in the literature (see e.g. [4, 5, 11, 19]) partly, because the measurements are sparse and the dependence between them are negligible. Assumption 1 excludes such a negligible temporal dependence to simplify the results. Assumption 2(i) requires the restriction boundary conditions of **x**_*it*_. Assumption 2(ii) assures that $E[{\tilde{\text{x}}}_{it}{\tilde{\text{x}}}_{it}^{\prime }]$ are bounded uniformly across *i*. Assumption 3 restricts the compactness on the parameter space and the inequality is important for the parameter’s identification. It corresponds to Condition 3 of [4, 20]. Assumption 4 is a mild regularity condition that is typically imposed in the quantile regression analysis. Assumption 4 restricts the smoothness and the boundedness of the density and its derivatives.

In applications, the variance-covariance matrices are unknown and need to be estimated. When *T* and *N* tend to infinity, we impose the following assumption.

**Assumption 5**: ${\hat{\text{V}}}_{i}={\text{V}}_{i}+o_{p}\left(1\right)$ as *T* → ∞. Assume that ${\left(N^{-1}\sum_{i=1}^{N}{\text{V}}_{i}^{-1}\right)}^{-1}\to \text{V}$, where $\text{V}≔\underset{N\to \infty }{\text{lim}}{\left(N^{-1}\sum_{i=1}^{N}{\text{V}}_{i}^{-1}\right)}^{-1}$ exists and is nonsingular.

**Assumption 5’**: ${\hat{\text{V}}}_{i}={\text{V}}_{i}+O_{p}\left(T^{-1/2}h_{N}^{-1/2}\right)$ for some *h*_*N*_ ↓ 0 uniformly across *i* and $\underset{N\to \infty }{\text{lim}}\;\frac{N}{Th_{N}}=0$ as *N* → ∞, *h*_*N*_ is a bandwidth. Assume that ${\left(N^{-1}\sum_{i=1}^{N}{\text{V}}_{i}^{-1}\right)}^{-1}\to \text{V}$, where $\text{V}≔\underset{N\to \infty }{\text{lim}}{\left(N^{-1}\sum_{i=1}^{N}{\text{V}}_{i}^{-1}\right)}^{-1}$ exists and is nonsingular.

An example that satisfies Assumption 5 is given in Equation 9 in [5]. Besides, an example satisfying Assumption 5’ is

$$\begin{array}{c} \tau \left(1-\tau \right)(\frac{1}{T}\sum_{t=1}^{T}K_{h_{N}}({\hat{u}}_{it}){\text{x}}_{it}{\text{x}}_{it}^{\prime }{)}^{-1}\frac{1}{T}\sum_{t=1}^{T}{\text{x}}_{it}{\text{x}}_{it}^{\prime }(\frac{1}{T}\sum_{t=1}^{T}K_{h_{N}}({\hat{u}}_{it}){\text{x}}_{it}{\text{x}}_{it}^{\prime }{)}^{-1}, \end{array}$$ (12)

where ${\hat{u}}_{it}=y_{it}-{\hat{\eta }}_{i}-{\text{z}}_{i}^{\prime }\hat{\gamma }-{\text{x}}_{it}^{\prime }\hat{\beta }$ and $K_{h_{N}}\left(\cdot \right)$ is defined in [19].

### Asymptotic properties of the W-QR estimator

**Assumption 6**: (i) The time-invariant regressors, **z**_*i*_ are independent of $v_{j}\left(\tau \right)=\eta_{j}\left(\tau \right)+{\bar{\varepsilon }}_{j}\left(\tau \right)$ for all *τ* ∈ *U* and *i*, *j* = 1, ⋯, *N*, and *η*_*i*_ and ${\bar{\varepsilon }}_{i}$ are independent. (ii) For all *i* = 1, ⋯, *N*, **z**_*i*_ satisfy the moment conditions $\|{\tilde{\text{z}}}_{i}\|<C_{M}$. (iii) As *N* → ∞, $N^{-1}\sum_{i=1}^{N}E[{\tilde{\text{z}}}_{i}{\tilde{\text{z}}}_{i}^{\prime }]\to {\text{Q}}_{\text{zz}}$.

**Assumption 7**: (i) For all *i* = 1, ⋯, *N*, $E[{\text{sup}}_{\tau \in U}|\eta_{i}\left(\tau \right){|}^{4+c_{M}}]\leq C_{M}$. (ii) As *N* → ∞, $N^{-1}\sum_{i=1}^{N}E[\eta_{i}(\tau )\eta_{i}(\tau ){\tilde{\text{z}}}_{i}{\tilde{\text{z}}}_{i}^{\prime }]\to J\left(\tau \right)$.

Assumption 6(i)-(ii) require that **z**_*i*_ is exogenous and bounded. Assumption 6(iii) and Assumption 7 are familiar identification conditions in regression analysis.

Under both sequential and simultaneous limits, we show the consistency of ${\hat{\gamma }}_{W-QR}\left(\tau \right)$ and ${\hat{\beta }}_{W-QR}\left(\tau \right)$.

**Theorem 1**. 1. Under Assumptions 1–3 and Assumptions 5–7,

$${\hat{\gamma }}_{W-QR}\left(\tau \right)\overset{p}{\to }\gamma \left(\tau \right),$$

$${\hat{\beta }}_{W-QR}\left(\tau \right)\overset{p}{\to }\beta \left(\tau \right)$$

as (*T*, *N*)_*seq*_ → ∞.

2. Under Assumptions 1–4, 5’and Assumptions 6–7,

$${\hat{\gamma }}_{W-QR}\left(\tau \right)\overset{p}{\to }\gamma \left(\tau \right),$$

$${\hat{\beta }}_{W-QR}\left(\tau \right)\overset{p}{\to }\beta \left(\tau \right)$$

as (*T*, *N*) → ∞ and $\frac{\text{log}\;N}{T}\to 0$.

Next, under the simultaneous limits, we show the asymptotic normality of ${\hat{\gamma }}_{W-QR}\left(\tau \right)$ and ${\hat{\beta }}_{W-QR}\left(\tau \right)$.

**Theorem 2**. Under Assumptions 1–4, 5’and Assumptions 6–7, as (*T*, *N*)→∞ and $\frac{N^{2}\left(\text{log}\;N\right)}{T}{\left|\text{log}\frac{{\left(\text{log}\;N\right)}^{0.5}}{T^{0.5}}\right|}^{2}\to 0$,

$$\sqrt{N}\left({\hat{\gamma }}_{W-QR}\left(\tau \right)-\gamma \left(\tau \right)\right)\overset{d}{\to }N\left(0,\Omega \left(\tau \right)\right),$$

and

$$\sqrt{NT}\left({\hat{\beta }}_{W-QR}\left(\tau \right)-\beta \left(\tau \right)\right)\overset{d}{\to }N\left(0,\text{V}\right),$$

where $\Omega \left(\tau \right)={\text{Q}}_{\text{zz}}J\left(\tau \right){\text{Q}}_{\text{zz}}^{\prime }$, **Q**_**zz**_ is defined in Assumption 6(iii) and *J*(*τ*) is defined in Assumption 7(ii), and **V** is defined in Assumption 5’.

### Asymptotic properties of the W-IVQR estimator

In this section, we study the asymptotic properties of the W-IVQR estimator. The W-IVQR estimator is proposed to deal with the case where **z**_*i*_ is endogenous variable. Assume there exists instrument **r**_*i*_ for time-invariant **z**_*i*_, where **r**_*i*_ is independent of *η*_*j*_(*τ*) and *ε*_*j*_(*τ*) for all *i* and *j* and *s* ≥ *p*. Throughout this section we will make the following assumptions.

**Assumption 6’**: As *N* → ∞,

$$\sum_{i=1}^{N}E\left[{\tilde{\text{z}}}_{i}{\tilde{\text{r}}}_{i}^{\prime }\right]\to {\text{Q}}_{\text{zr}},\;\sum_{i=1}^{N}E\left[{\tilde{\text{r}}}_{i}{\tilde{\text{r}}}_{i}^{\prime }\right]\to {\text{Q}}_{\text{rr}}.$$

**Assumption 7’**: (i) For all *τ* ∈ *U* and *i*, *j* = 1, ⋯, *N*, *E*[**r**_*i*_*η*_*j*_(*τ*)] = 0. (ii) As *N* → ∞, $N^{-1}\sum_{i=1}^{N}E[\eta_{i}(\tau )\eta_{i}(\tau ){\tilde{\text{r}}}_{i}{\tilde{\text{r}}}_{i}^{\prime }]\to \tilde{J}\left(\tau \right)$. (iii) For all *i* = 1, ⋯, *N* and *t* = 1, ⋯, *T*, *y*_*it*_ is independent of **r**_*i*_ conditional on (**x**_*it*_, **z**_*i*_, *η*_*i*_). (iv) For all *i* = 1, ⋯, *N*, $E[\|\tilde{{\text{r}}_{i}}{\|}^{4+c_{M}}]\leq C_{M}$.

Assumptions 6’-7’ are identification conditions.

**Theorem 3**. 1. Under Assumptions 1–3, 5 and 6’-7’,

$${\hat{\gamma }}_{W-IVQR}\left(\tau \right)\overset{p}{\to }\gamma \left(\tau \right),$$

$${\hat{\beta }}_{W-IVQR}\left(\tau \right)\overset{p}{\to }\beta \left(\tau \right)$$

as (*T*, *N*)_*seq*_ → ∞.

2. Under Assumptions 1–4, 5’-7’,

$${\hat{\gamma }}_{W-IVQR}\left(\tau \right)\overset{p}{\to }\gamma \left(\tau \right),$$

$${\hat{\beta }}_{W-IVQR}\left(\tau \right)\overset{p}{\to }\beta \left(\tau \right)$$

as (*T*, *N*) → ∞ and $\frac{\text{log}\;N}{T}\to 0$.

**Theorem 4**. Under Assumptions 1–4, 5’-7’, as (*T*, *N*) → ∞ and $\frac{N^{2}\left(\text{log}\;N\right)}{T}{\left|\text{log}\frac{{\left(\text{log}\;N\right)}^{0.5}}{T^{0.5}}\right|}^{2}\to 0$,

$$\sqrt{N}\left({\hat{\gamma }}_{W-IVQR}\left(\tau \right)-\gamma \left(\tau \right)\right)\overset{d}{\to }N\left(0,\tilde{\Omega }\left(\tau \right)\right),$$

and

$$\sqrt{NT}\left({\hat{\beta }}_{W-IVQR}\left(\tau \right)-\beta \left(\tau \right)\right)\overset{d}{\to }N\left(0,\text{V}\right),$$

where $\tilde{\Omega }\left(\tau \right)=\text{S}\tilde{J}\left(\tau \right){\text{S}}^{\prime }$, $\text{S}=({\text{Q}}_{\text{zr}}{\text{Q}}_{\text{rr}}^{-1}{\text{Q}}_{\text{zr}}^{\prime }{)}^{-1}{\text{Q}}_{\text{zr}}{\text{Q}}_{\text{rr}}^{-1}$, **Q**_**zr**_ and **Q**_**rr**_ are defined in Assumption 6’, $\tilde{J}\left(\tau \right)$ is defined in Assumption 7’(ii), and **V** defined in Assumption 5’.

Remark 4: In our treatment of panel data models we have assumed that a balanced panel is available, that is each cross-sectional unit has the same time periods available. Often, some periods are missing for some units, and we are left with an unbalanced panel. The two proposed estimators for unbalanced panel data are consistency and asympotic normality under specific assumptions. Follow [21], let ***κ***_*i*_ = (*κ*_*i*1_, ⋯, *κ*_*iT*_)′ denotes the *T* × 1 vector of selection indicators: *κ*_*it*_ ≡ 1 if (**x**_*it*_, *y*_*it*_) is observed, and zero otherwise. Consider the case ***κ***_*i*_ is independent of (***ε***_*i*_(*τ*), **x**_*i*_, **z**_*i*_, *η*_*i*_(*τ*)) for *τ* ∈ *U*, where **x**_*i*_ = (**x**_*i*1_, ⋯, **x**_*iT*_)′, ***ε***_*i*_ = (*ε*_*i*1_, ⋯, *ε*_*iT*_)′, the proposed estimators are consistency and asympotic normality by strengthening some assumptions, such as $\underset{i}{\text{min}}\;T_{i}$ satisfies the assumptions of *T*, where $T_{i}=\sum_{t=1}^{T}\kappa_{it}$. A more complicated problem arises when ***κ***_*i*_ depends on (***ε***_*i*_(*τ*), **x**_*i*_, **z**_*i*_, *η*_*i*_(*τ*)), for example, when *T*_*i*_ is treated as nonrandom more assumptions need to be modified.

## Monte Carlo simulation

We conduct some simulations to assess the finite sample performance of the proposed estimators, W-QR and W-IVQR estimator. We employ two variants of the data generating process (DGP). DGP A considers the time-invariant variable is exogenous. While under DGP B, the time-invariant variable is correlated with the fixed effects. Specifically, *y*_*it*_, fixed effect *α*_*i*_ and time-varying regressors are generated from the following model:

$$y_{it}=1+\alpha_{i}+\beta_{1}x_{it,1}+\beta_{2}x_{it,2}+\gamma_{1}z_{i1}+\gamma_{2}z_{i2}+\varepsilon_{it},\;i=1,2,\ldots ,N,t=1,2,\ldots ,T,$$

$$\alpha_{i}=w_{it,1}+w_{it,2}+\eta_{i}+0.1,$$

$$x_{it,1}=\eta_{i}g_{1t}+w_{it,1},$$

$$x_{it,2}=\eta_{i}g_{2t}+w_{it,2},$$

where *β*_1_ = *β*_2_ = *γ*_1_ = *γ*_2_ = 1, *g*_1*t*_ ∼ U(1, 2), *g*_2*t*_ ∼ U(1, 2), *η*_*i*_ ∼ 0.5(*χ*^2^(2) − 2) and $w_{it,1},w_{it,2}\overset{iid}{∼}N\left(0,1\right)$.

As regards time-invariant variables, *z*_*i*,*j*_ for *j* = 1, 2, we consider two different forms of generation of two time-invariant variables. Two time-invariant regressors *z*_*i*1_ and *z*_*i*2_ are generated as

$${\text{z}}_{i}=(\begin{array}{c} 1\\ 1 \end{array})+\Lambda {\bar{\text{w}}}_{i}+\alpha_{i}\phi +\zeta_{i},$$

where ${\text{z}}_{i}=(z_{i1},z_{i2}{)}^{\prime },{\bar{\text{w}}}_{i}=({\bar{w}}_{i1},{\bar{w}}_{i2}{)}^{\prime }$, ${\bar{w}}_{ij}=T^{-1}\sum_{t=1}^{T}w_{it,j}$, $\Lambda =(\begin{array}{cc} 1 & 1\\ 1 & 1 \end{array})$ and $\zeta_{i}\overset{iid}{∼}N(0,{\text{I}}_{2})$. In DGP A, we set ***ϕ*** = (*ϕ*_1_, *ϕ*_2_)′ = 0, where the time-invariant regressors are exogenous. While ***ϕ*** = (*ϕ*_1_, *ϕ*_2_)′ = (1, 1)′ is set in DGP B, meaning that the time-invariant regressors are correlated with fixed effects. For the instrument variables **r**_*i*_ used in DGP B are generated as

$${\text{r}}_{i}=\gamma_{\zeta }\zeta_{i}+{\text{T}}_{\text{w}}{\bar{\text{w}}}_{i}+\xi_{i},$$

with

$$\gamma_{\zeta }=(\begin{array}{cc} 1 & 0\\ 1 & 0\\ 0 & 1\\ 0 & 1 \end{array}),$$

$${\text{T}}_{\text{w}}=(\begin{array}{cc} 1 & 1\\ 1 & 1\\ 1 & 1\\ 1 & 1 \end{array}),$$

and $\xi_{i}\overset{iid}{∼}N(0,{\text{I}}_{4})$.

For DGP A, we consider two different process for *ε*_*it*_:

Case 1: $\varepsilon_{it}\overset{iid}{∼}N\left(0,1\right),\;i=1,2,\ldots ,N;t=1,2,\ldots ,T.$

Case 2: $\varepsilon_{it}\overset{iid}{∼}t\left(3\right),\;i=1,2,\ldots ,N;t=1,2,\ldots ,T.$

And for the DGP B, we also consider two different process for *ε*_*it*_:

Case 3: $\varepsilon_{it}\overset{iid}{∼}N\left(0,1\right),\;i=1,2,\ldots ,N;t=1,2,\ldots ,T.$

Case 4: $\varepsilon_{it}\overset{iid}{∼}t\left(3\right),\;i=1,2,\ldots ,N;t=1,2,\ldots ,T.$

Here we set the number of replications to 1000. For the sake of comparing the performance and efficiency between different methods, we compare the Bias and RMSE of the following estimators: fixed effects quantile regression (FEQR) and penalized quantile regression (PQR) as in Koenker [1] and Lamarche [2]; the grouped IVQR estimator (for short G. IV) of Chetverikov et al. [16]; the proposed W-QR estimator and the proposed W-IVQR estimator. In the simulations, we report results considering {(*N*, *T*)} = {(50, 50), (50, 100), (100, 50), (100, 100)}, and *τ* ∈ {0.25, 0.5, 0.75}. Tables 1–4 report the estimation results for the DGP A. Tables 5–8 report the estimation results for the DGP B. The minimum values are marked in bold in each case in the table.

**Table 1:** Bias and RMSE of 3 estimators for *γ*_1_ and *γ*_2_ when $\varepsilon_{it}\overset{iid}{∼}N\left(0,1\right)$ in the DGP A.

	(*N*, *T*)	*τ*	*γ*_1_			*γ*_2_		
			FEQR	PQR	W-QR	FEQR	PQR	W-QR
Bias	(50,50)	0.25	-0.682	-0.209	**0.002**	-1.000	-0.790	**-0.004**
		0.5	-0.870	-0.187	**0.000**	-0.999	-0.807	**-0.001**
		0.75	-3.680	-0.186	**-0.005**	-1.001	-0.810	**0.007**
	(50,100)	0.25	0.261	-0.233	**0.005**	-1.001	-0.764	**0.006**
		0.5	-1.011	-0.229	**-0.007**	-1.000	-0.783	**0.008**
		0.75	-1.310	-0.191	**0.009**	-1.000	-0.801	**0.002**
	(100,50)	0.25	-0.160	-0.223	**0.005**	-0.998	-0.775	**-0.002**
		0.5	-0.505	-0.223	**-0.006**	-1.001	-0.780	**0.000**
		0.75	-8.610	-0.240	**0.002**	-1.002	-0.762	**0.004**
	(100,100)	0.25	-3.594	-0.250	**-0.001**	-0.999	-0.747	**-0.001**
		0.5	-27.668	-0.234	**0.002**	-1.002	-0.768	**-0.001**
		0.75	-1.293	-0.248	**-0.001**	-0.999	-0.761	**-0.001**
RMSE	(50,50)	0.25	7.538	0.498	**0.156**	1.000	0.895	**0.153**
		0.5	5.513	0.495	**0.160**	0.999	0.915	**0.164**
		0.75	65.202	0.488	**0.159**	1.002	0.908	**0.160**
	(50,100)	0.25	25.520	0.536	**0.153**	1.002	0.887	**0.152**
		0.5	2.580	0.539	**0.155**	1.001	0.912	**0.154**
		0.75	3.808	0.466	**0.145**	1.001	0.895	**0.159**
	(100,50)	0.25	16.673	0.536	**0.109**	0.999	0.910	**0.110**
		0.5	5.331	0.529	**0.111**	1.002	0.905	**0.110**
		0.75	188.145	0.533	**0.107**	1.003	0.891	**0.114**
	(100,100)	0.25	63.234	0.554	**0.105**	1.000	0.893	**0.108**
		0.5	651.733	0.546	**0.107**	1.006	0.911	**0.103**
		0.75	8.609	0.557	**0.101**	1.003	0.899	**0.105**

**Table 2:** Bias and RMSE of 3 estimators for *β*_1_ and *β*_2_ when $\varepsilon_{it}\overset{iid}{∼}N\left(0,1\right)$ in the DGP A.

	(*N*, *T*)	*τ*	*β*_1_			*β*_2_		
			FEQR	PQR	W-QR	FEQR	PQR	W-QR
Bias	(50,50)	0.25	**0.000**	0.023	**0.000**	**0.000**	0.023	-0.001
		0.5	**-0.001**	0.024	**-0.001**	**-0.001**	0.023	-0.003
		0.75	**0.001**	0.025	**0.001**	-0.001	0.024	**0.000**
	(50,100)	0.25	**0.000**	0.013	-0.001	**0.000**	0.013	**0.000**
		0.5	**0.001**	0.013	**0.001**	**0.000**	0.013	**0.000**
		0.75	**0.000**	0.013	**0.000**	**0.001**	0.013	**0.000**
	(100,50)	0.25	**0.000**	0.025	-0.001	**0.000**	0.024	-0.001
		0.5	**0.000**	0.024	-0.001	**-0.001**	0.023	-0.002
		0.75	**0.001**	0.026	0.003	**0.000**	0.025	0.001
	(100,100)	0.25	0.001	0.013	**0.000**	**0.000**	0.013	**0.000**
		0.5	**0.000**	0.012	**0.000**	**0.000**	0.013	0.001
		0.75	-0.001	0.012	**0.000**	**0.000**	0.013	0.001
RMSE	(50,50)	0.25	**0.019**	0.031	0.034	**0.020**	0.031	0.035
		0.5	**0.020**	0.032	0.039	**0.019**	0.030	0.037
		0.75	**0.019**	0.032	0.039	**0.019**	0.031	0.038
	(50,100)	0.25	**0.013**	0.019	0.025	**0.014**	0.019	0.026
		0.5	**0.014**	0.019	0.023	**0.014**	0.019	0.023
		0.75	**0.014**	0.019	0.028	**0.013**	0.019	0.025
	(100,50)	0.25	**0.013**	0.029	0.026	**0.014**	0.028	0.026
		0.5	**0.014**	0.028	0.025	**0.014**	0.028	0.025
		0.75	**0.013**	0.030	0.028	**0.014**	0.029	0.028
	(100,100)	0.25	**0.010**	0.017	0.018	**0.010**	0.016	0.018
		0.5	**0.010**	0.016	0.017	**0.010**	0.017	0.017
		0.75	**0.010**	0.016	0.018	**0.010**	0.016	0.018

**Table 3:** Bias and RMSE of 3 estimators for *γ*_1_ and *γ*_2_ when $\varepsilon_{it}\overset{iid}{∼}t\left(3\right)$ in the DGP A.

	(*N*, *T*)	*τ*	*γ*_1_			*γ*_2_		
			FEQR	PQR	W-QR	FEQR	PQR	W-QR
Bias	(50,50)	0.25	-0.973	-0.192	**0.007**	-1.001	-0.811	**0.001**
		0.5	-1.037	-0.177	**-0.001**	-0.998	-0.822	**-0.003**
		0.75	-1.053	-0.204	**0.013**	-1.000	-0.784	**0.000**
	(50,100)	0.25	-0.900	-0.204	**-0.009**	-1.002	-0.800	**0.002**
		0.5	-0.184	-0.220	**-0.005**	-0.998	-0.785	**-0.010**
		0.75	-1.086	-0.195	**0.000**	-1.004	-0.808	**0.007**
	(100,50)	0.25	-0.757	-0.240	**0.003**	-0.999	-0.763	**0.002**
		0.5	-0.839	-0.226	**0.005**	-1.000	-0.776	**0.003**
		0.75	-0.834	-0.248	**-0.001**	-0.999	-0.752	**0.002**
	(100,100)	0.25	-0.929	-0.246	**-0.001**	-1.000	-0.761	**-0.001**
		0.5	-0.639	-0.239	**-0.001**	-1.000	-0.752	**0.000**
		0.75	-0.928	-0.243	**0.002**	-1.001	-0.758	**-0.001**
RMSE	(50,50)	0.25	5.164	0.503	**0.165**	1.001	0.919	**0.166**
		0.5	9.801	0.475	**0.159**	0.999	0.921	**0.158**
		0.75	8.138	0.522	**0.161**	1.000	0.905	**0.164**
	(50,100)	0.25	4.283	0.492	**0.151**	1.003	0.904	**0.153**
		0.5	13.902	0.539	**0.152**	0.999	0.915	**0.149**
		0.75	1.836	0.498	**0.147**	1.005	0.910	**0.147**
	(100,50)	0.25	5.320	0.548	**0.116**	1.000	0.898	**0.113**
		0.5	6.653	0.533	**0.107**	1.001	0.912	**0.114**
		0.75	6.764	0.556	**0.116**	1.001	0.894	**0.121**
	(100,100)	0.25	2.842	0.564	**0.108**	1.002	0.908	**0.111**
		0.5	9.194	0.553	**0.106**	1.004	0.898	**0.105**
		0.75	4.804	0.543	**0.109**	1.002	0.892	**0.109**

**Table 4:** Bias and RMSE of 3 estimators for *β*_1_ and *β*_2_ when $\varepsilon_{it}\overset{iid}{∼}\;t\left(3\right)$ in the DGP A.

	(*N*, *T*)	*τ*	*β*_1_			*β*_2_		
			FEQR	PQR	W-QR	FEQR	PQR	W-QR
Bias	(50,50)	0.25	**0.000**	0.023	-0.002	**0.001**	0.025	0.004
		0.5	**0.003**	0.025	**0.003**	**-0.001**	0.022	0.002
		0.75	-0.003	0.021	**-0.002**	**0.001**	0.025	0.004
	(50,100)	0.25	0.002	0.014	**0.001**	**0.000**	0.012	0.001
		0.5	**0.000**	0.012	**0.000**	**0.000**	0.012	**0.000**
		0.75	**0.000**	0.012	**0.000**	**-0.001**	0.012	**-0.001**
	(100,50)	0.25	0.001	0.026	**0.000**	**-0.001**	0.024	-0.002
		0.5	**0.001**	0.025	0.002	**0.000**	0.023	0.001
		0.75	0.001	0.026	**0.000**	**0.000**	0.024	0.001
	(100,100)	0.25	**0.000**	0.013	-0.001	**0.000**	0.013	**0.000**
		0.5	**0.001**	0.014	**0.001**	**0.000**	0.012	**0.000**
		0.75	**0.000**	0.013	0.002	**0.000**	0.012	0.001
RMSE	(50,50)	0.25	**0.034**	0.041	0.044	**0.034**	0.042	0.045
		0.5	**0.032**	0.040	0.039	**0.033**	0.040	0.038
		0.75	**0.030**	0.037	0.044	**0.034**	0.041	0.046
	(50,100)	0.25	**0.022**	0.026	0.030	**0.024**	0.027	0.032
		0.5	**0.023**	0.026	0.024	**0.024**	0.026	0.025
		0.75	**0.025**	0.028	0.031	**0.025**	0.027	0.032
	(100,50)	0.25	**0.024**	0.035	0.035	**0.023**	0.033	0.035
		0.5	**0.024**	0.035	0.027	**0.023**	0.033	0.027
		0.75	**0.024**	0.035	0.034	**0.025**	0.035	0.033
	(100,100)	0.25	**0.017**	0.021	0.023	**0.018**	0.022	0.024
		0.5	**0.017**	0.021	0.018	**0.016**	0.020	0.018
		0.75	**0.016**	0.021	0.023	**0.017**	0.021	0.022

**Table 5:** Bias and RMSE of 4 estimators for *γ*_1_ and *γ*_2_ when $\varepsilon_{it}\overset{iid}{∼}N\left(0,1\right)$ in the DGP B.

	(*N*, *T*)	*τ*	*γ*_1_				*γ*_2_			
			FEQR	PQR	G. IV	W-IVQR	FEQR	PQR	G. IV	W-IVQR
Bias	(50,50)	0.25	-1.402	-0.221	0.001	**0.000**	-1.000	-0.781	**0.002**	0.006
		0.5	-0.410	-0.202	-0.005	**-0.004**	-0.999	-0.805	**0.003**	**0.003**
		0.75	-0.361	-0.206	**0.003**	0.006	-1.000	-0.793	**-0.001**	-0.003
	(50,100)	0.25	-1.669	-0.223	**0.002**	-0.003	-1.001	-0.761	**0.005**	**0.005**
		0.5	-1.033	-0.220	**0.001**	**-0.001**	-0.999	-0.779	**0.004**	**0.004**
		0.75	-1.341	-0.193	-0.009	**-0.005**	-1.001	-0.800	0.004	**0.001**
	(100,50)	0.25	-0.964	-0.221	**0.004**	0.007	-1.000	-0.775	**0.000**	0.001
		0.5	-1.121	-0.261	**0.001**	0.003	-1.005	-0.746	**0.002**	**0.002**
		0.75	-1.244	-0.261	-0.006	**-0.005**	-0.998	-0.741	**-0.001**	**-0.001**
	(100,100)	0.25	-1.371	-0.205	-0.005	**-0.003**	-0.999	-0.790	-0.005	**-0.002**
		0.5	-1.585	-0.244	0.009	**0.003**	-1.000	-0.753	**0.002**	**0.002**
		0.75	-1.218	-0.204	-0.008	**-0.004**	-1.000	-0.797	**-0.003**	**-0.003**
RMSE	(50,50)	0.25	16.969	0.519	**0.200**	0.230	1.000	0.902	**0.203**	**0.203**
		0.5	15.556	0.500	**0.196**	0.228	1.001	0.915	**0.201**	0.204
		0.75	41.039	0.514	**0.202**	0.231	1.001	0.904	**0.190**	**0.190**
	(50,100)	0.25	15.890	0.534	**0.207**	0.232	1.002	0.894	**0.190**	**0.190**
		0.5	2.714	0.519	**0.182**	0.214	1.002	0.908	0.184	**0.183**
		0.75	7.619	0.507	**0.192**	0.219	1.002	0.906	**0.183**	0.186
	(100,50)	0.25	9.370	0.550	**0.132**	0.154	1.001	0.919	**0.132**	**0.132**
		0.5	3.421	0.571	**0.131**	0.153	1.007	0.899	**0.132**	**0.132**
		0.75	9.869	0.588	**0.134**	0.156	0.998	0.902	**0.143**	**0.143**
	(100,100)	0.25	8.636	0.490	**0.128**	0.157	1.001	0.903	**0.127**	0.128
		0.5	9.598	0.557	**0.130**	0.152	1.004	0.902	**0.136**	**0.136**
		0.75	3.217	0.490	**0.128**	0.146	1.003	0.906	**0.136**	0.138

**Table 6:** Bias and RMSE of 3 estimators for *β*_1_ and *β*_2_ when $\varepsilon_{it}\overset{iid}{∼}N\left(0,1\right)$ in the DGP B.

	(*N*, *T*)	*τ*	*β*_1_			*β*_2_		
			FEQR	PQR	W-IVQR	FEQR	PQR	W-IVQR
Bias	(50,50)	0.25	**0.000**	0.024	-0.002	**-0.001**	0.024	-0.002
		0.5	**0.000**	0.023	-0.001	**-0.001**	0.023	-0.003
		0.75	-0.002	0.022	**0.000**	**-0.001**	0.023	**-0.001**
	(50,100)	0.25	**-0.001**	0.012	-0.002	**-0.001**	0.012	**-0.001**
		0.5	**0.000**	0.013	**0.000**	**0.000**	0.012	**0.000**
		0.75	-0.001	0.011	**0.000**	**0.001**	0.013	0.002
	(100,50)	0.25	0.001	0.025	**0.000**	**0.000**	0.024	-0.001
		0.5	**0.000**	0.025	**0.000**	**0.000**	0.025	0.001
		0.75	**0.000**	0.024	0.002	**-0.001**	0.023	**-0.001**
	(100,100)	0.25	**0.000**	0.012	**0.000**	**0.000**	0.013	**0.000**
		0.5	**0.000**	0.013	**0.000**	**0.000**	0.013	**0.000**
		0.75	**0.000**	0.013	**0.000**	**0.000**	0.013	**0.000**
RMSE	(50,50)	0.25	**0.020**	0.032	0.038	**0.018**	0.032	0.036
		0.5	**0.019**	0.031	0.033	**0.019**	0.031	0.034
		0.75	**0.020**	0.030	0.037	**0.019**	0.032	0.037
	(50,100)	0.25	**0.013**	0.019	0.026	**0.013**	0.018	0.026
		0.5	**0.013**	0.018	0.022	**0.013**	0.019	0.023
		0.75	**0.014**	0.019	0.025	**0.013**	0.019	0.025
	(100,50)	0.25	**0.014**	0.029	0.029	**0.014**	0.028	0.028
		0.5	**0.014**	0.029	0.026	**0.013**	0.029	0.025
		0.75	**0.015**	0.028	0.029	**0.013**	0.028	0.027
	(100,100)	0.25	**0.010**	0.016	0.019	**0.010**	0.016	0.019
		0.5	**0.010**	0.016	0.017	**0.010**	0.016	0.017
		0.75	**0.010**	0.017	0.019	**0.009**	0.016	0.019

**Table 7:** Bias and RMSE of 4 estimators for *γ*_1_ and *γ*_2_ when $\varepsilon_{it}\overset{iid}{∼}t\left(3\right)$ in the DGP B.

	(*N*, *T*)	*τ*	*γ*_1_				*γ*_2_			
			FEQR	PQR	G. IV	W-IVQR	FEQR	PQR	G. IV	W-IVQR
Bias	(50,50)	0.25	-1.999	-0.228	**-0.007**	-0.009	-1.000	-0.778	**-0.004**	**-0.004**
		0.5	-0.212	-0.227	**0.003**	0.009	-1.001	-0.784	**0.005**	**0.005**
		0.75	-1.136	-0.225	**-0.001**	**0.001**	-1.002	-0.771	**-0.004**	**-0.004**
	(50,100)	0.25	-0.891	-0.220	0.003	**-0.001**	-0.999	-0.787	-0.003	**-0.002**
		0.5	-1.596	-0.175	-0.008	**-0.007**	-0.996	-0.809	0.007	**0.007**
		0.75	-1.513	-0.179	-0.005	**0.001**	-1.000	-0.814	0.003	**0.002**
	(100,50)	0.25	-0.186	-0.244	-0.009	**-0.006**	-1.001	-0.754	**0.001**	0.002
		0.5	-1.100	-0.214	**-0.004**	**-0.004**	-1.000	-0.780	**-0.006**	**-0.006**
		0.75	-0.512	-0.246	**-0.002**	-0.006	-1.001	-0.767	**-0.004**	**-0.004**
	(100,100)	0.25	-0.492	-0.195	**0.000**	-0.001	-1.000	-0.806	**-0.002**	**-0.002**
		0.5	2.895	-0.193	**-0.001**	**0.001**	-0.996	-0.810	**-0.002**	**-0.002**
		0.75	0.126	-0.205	**-0.002**	**0.002**	-1.001	-0.795	-0.004	**-0.003**
RMSE	(50,50)	0.25	16.016	0.533	**0.204**	0.235	1.000	0.899	**0.205**	**0.205**
		0.5	21.622	0.512	**0.192**	0.217	1.001	0.909	**0.201**	0.202
		0.75	4.836	0.534	**0.203**	0.231	1.003	0.904	**0.219**	0.220
	(50,100)	0.25	3.330	0.521	**0.206**	0.231	1.003	0.898	**0.188**	0.190
		0.5	14.828	0.461	**0.186**	0.217	0.998	0.907	**0.190**	0.192
		0.75	9.347	0.484	**0.202**	0.233	1.000	0.917	**0.187**	0.190
	(100,50)	0.25	17.432	0.535	**0.136**	0.160	1.001	0.887	**0.139**	**0.139**
		0.5	2.566	0.509	**0.138**	0.165	1.002	0.903	**0.137**	0.138
		0.75	14.013	0.542	**0.142**	0.169	1.003	0.899	**0.146**	**0.146**
	(100,100)	0.25	11.642	0.472	**0.133**	0.152	1.003	0.905	**0.129**	0.128
		0.5	71.825	0.506	**0.127**	0.153	0.997	0.926	**0.133**	**0.133**
		0.75	25.651	0.505	**0.133**	0.155	1.003	0.910	**0.143**	0.144

**Table 8:** Bias and RMSE of 3 estimators for *β*_1_ and *β*_2_ when $\varepsilon_{it}\overset{iid}{∼}t\left(3\right)$ in the DGP B.

	(*N*, *T*)	*τ*	*β*_1_			*β*_2_		
			FEQR	PQR	W-IVQR	FEQR	PQR	W-IVQR
Bias	(50,50)	0.25	**-0.003**	0.021	-0.004	**-0.001**	0.022	-0.002
		0.5	**0.001**	0.024	**0.001**	0.003	0.026	**0.001**
		0.75	**0.001**	0.025	-0.003	**0.002**	0.026	**0.002**
	(50,100)	0.25	**0.000**	0.012	**0.000**	**0.000**	0.012	-0.001
		0.5	**0.000**	0.013	-0.001	**-0.002**	0.011	-0.003
		0.75	**0.000**	0.013	**0.000**	**0.000**	0.013	0.001
	(100,50)	0.25	-0.002	0.023	**-0.001**	**0.000**	0.024	-0.002
		0.5	0.002	0.026	**0.000**	0.001	0.025	**0.000**
		0.75	**-0.001**	0.023	-0.002	0.001	0.025	**0.000**
	(100,100)	0.25	**-0.001**	0.012	**-0.001**	**0.001**	0.014	**0.001**
		0.5	**0.000**	0.012	-0.001	**0.000**	0.013	**0.000**
		0.75	0.001	0.014	**0.000**	**-0.001**	0.012	**0.001**
RMSE	(50,50)	0.25	**0.033**	0.038	0.045	**0.032**	0.039	0.048
		0.5	**0.032**	0.040	0.038	**0.034**	0.042	0.038
		0.75	**0.034**	0.041	0.047	**0.033**	0.041	0.046
	(50,100)	0.25	**0.025**	0.028	0.030	**0.024**	0.027	0.033
		0.5	**0.022**	0.026	0.026	**0.024**	0.026	0.026
		0.75	**0.023**	0.027	0.033	**0.024**	0.027	0.032
	(100,50)	0.25	**0.025**	0.034	0.034	**0.025**	0.035	0.034
		0.5	**0.023**	0.034	0.026	**0.023**	0.034	0.027
		0.75	**0.024**	0.033	0.034	**0.024**	0.034	0.034
	(100,100)	0.25	**0.017**	0.020	0.022	**0.016**	0.021	0.023
		0.5	**0.016**	0.020	0.019	**0.017**	0.021	0.020
		0.75	**0.016**	0.021	0.023	**0.017**	0.021	0.025

Table 1 provides the Bias and RMSE of the three estimators for *γ*_1_ and *γ*_2_ when $\varepsilon_{it}\overset{iid}{∼}N\left(0,1\right)$ in the Case 1 of DGP A. It is clear that W-QR estimators are significantly better than the other two estimators. The W-QR estimator shows obvious advantages in Bias. The W-QR estimators is approximately unbiased, while the other two estimators are seriously biased. In terms of RMSE, the RMSE of the W-QR estimator are consistently smaller than those of the other two estimators in each quantile with the same sample size. It is noted that the RMSE of the FEQR estimator are very large. The simulation results indicate that, for the coefficients of time-invariant variables, the W-QR method can effectively increase precision of estimation.

Table 2 shows the Bias and RMSE of the three estimators for *β*_1_ and *β*_2_ when $\varepsilon_{it}\overset{iid}{∼}N\left(0,1\right)$ in the Case 1 of DGP A. We can see that the FEQR and W-QR estimators are approximately unbiased. The performance of W-QR estimator is slightly worse than that of FEQR in terms of RMSE, but the RMSE of the W-QR estimator reducing with the increase of *N* and *T*. On the other hand, because the Bias and RMSE of W-QR estimator for *γ*_1_ and *γ*_2_ are best, and the Bias for *β*_1_ and *β*_2_ are approximately unbiased, the W-QR estimator has the best overall performance. In addition, the FEQR estimator for *γ*_1_ and *γ*_2_ perform worst, and the PQR estimator for *β*_1_ and *β*_2_ perform worst.

The Bias and RMSE of the three estimators when $\varepsilon_{it}\overset{iid}{∼}\;t\left(3\right)$ in the Case 2 of DGP A are presented separately in Tables 3 and 4. Overall, they are similar to those when $\varepsilon_{it}\overset{iid}{∼}N\left(0,1\right)$. Considering *γ*_1_ and *γ*_2_, the Bias and RMSE of W-QR estimator are smaller than those of the other two estimators in each quantile with the same sample size. The W-QR estimator performs best. Considering *β*_1_ and *β*_2_, the W-QR estimators are approximately unbiased as *N* and *T* increase. Generally the W-QR estimator has the best overall performance.

Besides, we also find that the Bias and RMSE of for W-QR estimator decrease as sample size increases. The RMSE of the W-QR estimator for *γ*_1_ and *γ*_2_ decrease obviously with the increase of *N* but not *T*, as *γ*_1_ and *γ*_2_ are the coefficients of time-invariant variables. Meanwhile, the Bias and RMSE of the W-QR estimator for *β*_1_ and *β*_2_ decrease obviously as *T* increases but not as *N* increases because of the incidental parameter problem.

Table 5 gives the Bias and RMSE of the four estimators for *γ*_1_ and *γ*_2_ when $\varepsilon_{it}\overset{iid}{∼}N\left(0,1\right)$ in the Case 3 of DGP B. It can be seen that W-IVQR estimator and G. IV estimator among the four estimators are significantly better than the other two estimators in terms of Bias and RMSE. The Bias of the W-IVQR estimator and G. IV estimator are about in the three decimal places, while the Bias of other two are in single digits and decimal places. In terms of RMSE, the W-IVQR estimator and the G. IV estimator are also consistently smaller than the other two estimators in each quantile with the same sample size. The results of the G. IV estimator and the W-IVQR estimator are similar. The reason is that W-QR is the calculation result of the centralized 2SLS, and the G. IV estimator calculates the 2SLS. Compared with the G. IV estimator, the Bias of the W-IVQR estimator is generally smaller than that of the G. IV estimator, while the RMSE of W-IVQR estimator is slightly larger than that of the G. IV estimator.

Table 6 gives the Bias and RMSE of the three estimation methods for *β*_1_ and *β*_2_ when $\varepsilon_{it}\overset{iid}{∼}N\left(0,1\right)$ in the Case 3 of DGP B. Notice that the G. IV estimator only gives the estimation of *γ*_1_ and *γ*_2_, and does not give the estimation of *β*_1_ and *β*_2_. The estimation results shown in Table 6 are similar to those in Table 2. The W-IVQR and FEQR estimator are approximately unbiased. The performance of W-IVQR estimator is slightly worse than that of FEQR in terms of RMSE, but the RMSE of the W-IVQR estimator reduces as sample size increases. In general, the W-IVQR estimator has the best overall performance as the G. IV method cannot estimate *β*_1_ and *β*_2_, and the Bias of the W-IVQR eatimator for *β*_1_ and *β*_2_ are approximately unbiased.

Tables 7 and 8 separately give the estimation results when $\varepsilon_{it}\overset{iid}{∼}t\left(3\right)$ in the Case 4 of DGP B, which are similar to those of Tables 5 and 6. The W-IVQR estimator has the best overall performance. Regarding *γ*_1_ and *γ*_2_, in terms of Bias, the FEQR estimator and the PQR estimator perform poorly, and the W-IVQR and G. IV estimators are are approximately unbiased as *N* and *T* increase. The RMSE of W-IVQR estimator and G. IV estimator are closer to each other, which are much better than those of the FEQR estimator’s and the PQR estimator’s. Considering *β*_1_ and *β*_2_, the FEQR estimator and W-IVQR estimator are approximately unbiased. In addition, it can be seen that the Bias and RMSE of the W-IVQR estimator decrease as *T* and *N* increase. As both *N* and *T* become larger, the Bias and RMSE of the W-IVQR estimator become significantly smaller.

Furthermore, comparing the results of W-QR estimator in Tables 1–4 and W-IVQR estimator in Tables 5–8, it can be found that the estimation of *β*_1_ and *β*_2_ are not sensitive to whether the time-invariant covariates are endogenous or not. In other words, estimation accuracy of exogenous time-varying variables is not sensitive to instrumental variables.

From the above analysis, we can find that the W-QR estimator has absolute advantages over the FEQR and PQR estimators in estimating the coefficients of exogenous time-invariant covariates, and the W-IVQR and G. IV estimators perform much better than the other two estimators in estimating the coefficients of endogenous time-invariant covariates. What’s more, it is noted that the RMSE of G. IV’s estimator is less than W-IVQR in most cases. Meanwhile, the FEQR and the weighted estimators for the coefficients of time-varying covariates are asymptotically unbiased, and the RMSE of the FEQR estimator is uniformly smaller than the proposed estimators. A nature idea arises that for model (1), we can use the FEQR method to estimate ***β***, G. IV method to estimate ***γ***, so it seems that we can get a more robust estimator. However, the disadvantage of FEQR estimator is that it is time-consuming to solve a large optimization problem, and the asymptotic properties of G. IV estimator need to be modified. In sum, the W-QR and W-IVQR method can better estimate the static panel data model with time-invariant regressors.

## Application

### Model construction

The gravity model has been the cornerstone of empirical trade analysis since its inception. It is used to estimate the marginal effects of various trade determinants and to test hypothesized relationships. Traditional estimation methods derive marginal effects of covariates at the mean; alternatively, only estimate the mean effects of explanatory variables on trade flows. This study applies the new quantile approach with an application to analyze the effects of the influence factors of China’s exports using the trade gravity model. P*ö*yh*ö*nen [22] and Tibergen [23] first used the idea of the law of universal gravitation to explain the international trade flow, which received good empirical support in the study of practical problems, and then a large number of international trade-related studies began to use the trade gravity model, see e.g., Eaton and Kortum [24], Freeman and Lewis [25], Greaney and Kiyota [26]. The basic form of the trade gravity model can be expressed as

$$Y_{ij}=K\frac{X_{i}X_{j}}{D_{ij}^{2}},$$

where *Y*_*ij*_ is the total value of trade between country *i* and country *j*, *X*_*i*_ and *X*_*j*_ are the GDP of two countries respectively, *D*_*ij*_ represents the geographical distance between two countries, and *K* is a constant coefficient. It can be observed that the value of trade between the two countries are positively correlated with the gross national product of each country and negatively correlated with the geographical distance between the two countries. Meanwhile, the general trade gravity model can be written in the following form:

$$\begin{array}{c} Y_{ij}=K\frac{X_{i}^{\alpha }X_{j}^{\beta }}{D_{ij}^{\theta }}, \end{array}$$ (13)

where *α*, *β* and *γ* represent the elasticity coefficients of the gross national product *X*_*i*_, *X*_*j*_ and the geographical distance *D*_*ij*_ respectively. In fact, the coefficients *α*, *β* and *θ* capture the extent to which GDP and geographical distance affect the total trade between the two countries. Take logarithms of formula (13), we obtain

$$\begin{array}{c} \text{ln}\;Y_{ij}=\text{ln}\;K+\alpha \;\text{ln}\;X_{i}+\beta \;\text{ln}\;X_{j}+\gamma \;\text{ln}\;D_{ij}+\varepsilon_{ij}. \end{array}$$ (14)

In order to analyze China’s international trade exports as comprehensively as possible and to capture the dynamic heterogeneity of various influencing factors at different levels of trade volume, in this section we construct the above trade gravity model (14) using cross-country panel data of 98 countries or regions with which China has trade transactions for 29 years from 1990–2018, Export data dataset S1 Dataset. F test and Hausman test are carried out to determine the type of panel model for (14). As shown in Table 9, the p-value of F test is 0.000, which rejects the null hypothesis, that is, the fixed effect model is better than the mixed effect model. Hausman test is used to determine whether to build the fixed effect model or the random effect model. The p-value of the Hausman test from Table 10 is less than 0.05, which means that a fixed effect model should be built.

**Table 9:** F test.

F statistic	p-value
59.841	<2.2e-16

**Table 10:** Hausman test.

Chisq statistic	p-value
12.726	0.001724

Thus, (14) can be written as

$$\begin{array}{c} \text{ln}\;Export_{it}=C+\beta_{1}\text{ln}\;gdp_{it}+\beta_{2}\text{ln}\;Chngdp_{t}+\gamma \;\text{ln}\;D_{i}+\alpha_{i}+\varepsilon_{it}, \end{array}$$ (15)

where ln *Export*_*it*_ is the logarithm of China’s total trade exports to country *i* in year *t*, ln *gdp*_*it*_ and ln *Chngdp*_*t*_ denote respectively the logarithm of GDP of the *i*th country and China in year *t*, ln *D*_*i*_ indicates the logarithm of the distance between the Chinese capital and the capital of country *i*, *α*_*i*_ is unobserved individual fixed effect.

### Data sources

Data on trade exports and GDP are obtained from the International Monetary Fund (IMF) and World Bank Indicators (WDI), and the distances between capitals are obtained from the Centre d*é*tudes prospectives et d’informations internationals (CEPII) database. Besides, data used in the improved model contains the information about *APEC* and *DC* is from Baidu encyclopedia, area data comes from the Ministry of Foreign Affairs of the People’s Republic of China.

### Model estimation

**Fig 1:**
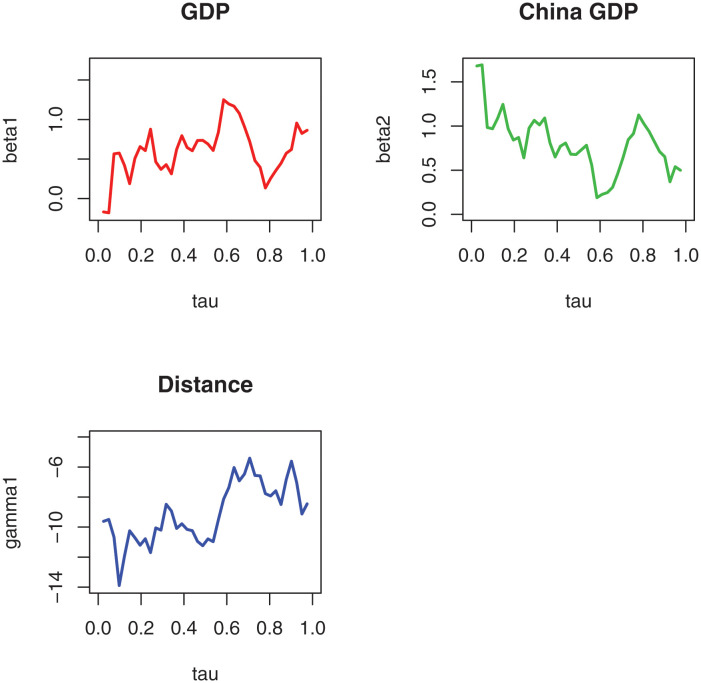
The W-QR estimators at different quantile.

The FEF method in [13] and the W-QR method are used here to solve (15) as the ln *D*_*i*_ here is time-invariant variable. We calculate the standard error of the W-QR estimators based on block bootstrap. The estimation results are presented in Table 11 and Fig 1. As seen in Table 11, the ${\hat{\beta }}_{1}$ and ${\hat{\beta }}_{2}$ are positive and $\hat{\gamma }$ are negative at the given quartiles, which are consistent with the estimation results of FEF. The results of W-QR method report more abundant estimation results which can capture fully the influences of covariates on the response, compared with the FEF method.

**Table 11:** The estimation results of W-QR estimator and FEF estimaor.

W-QR						FEF
	0.1	0.25	0.5	0.75	0.9	
*β*_1_	0.561	0.871	0.688	0.454	0.601	0.883
	(0.854)	(0.792)	(0.766)	(0.662)	(1.050)	(0.067)
*β*_2_	0.982	0.645	0.728	0.866	0.672	0.854
	(0.816)	(0.743)	(0.7284)	(0.600)	(0.938)	(0.037)
*γ*	-13.963	-11.660	-10.865	-6.453	-5.613	-0.852
	(6.796)	(5.038)	(3.947)	(4.186)	(5.205)	(0.255)

The value in bracket is standard error.

Moreover, from Fig 1, the ${\hat{\beta }}_{1}$ and ${\hat{\beta }}_{2}$ are positive at all quartiles, indicating that the GDP of other countries and China’s GDP play a role in boosting China’s total exports. The larger the GDP the greater the producer demand, and therefore will have a boosting effect on China’s export trade. $\hat{\gamma }$ are negative at all quartiles, indicating that the distance factor acts as a disincentive to China’s export trade, i.e., the trade risks and costs associated with the increased geographical distance between the two sides of the trade, which inhibits China’s export trade.

### Improved model with more time-invariant regressors and estimation

**Fig 2:**
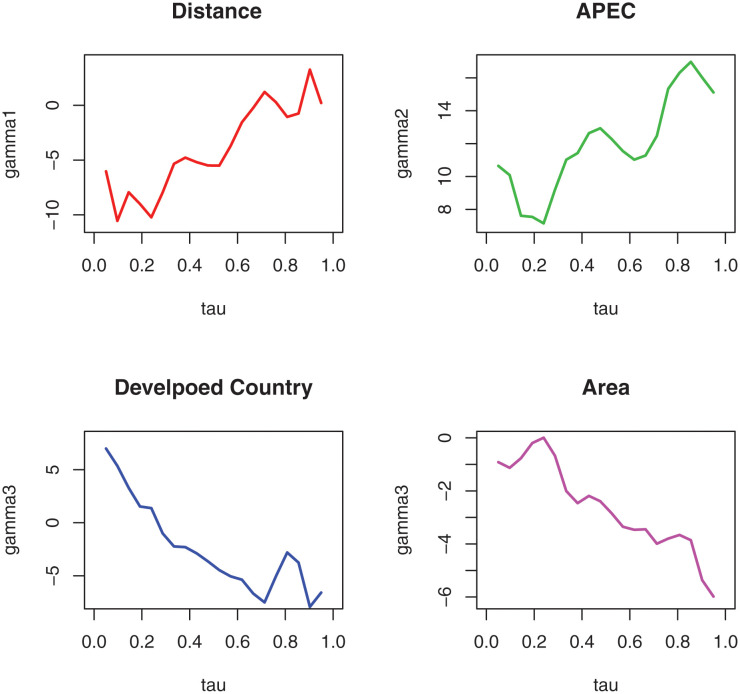
The W-QR estimators for time-invariant covariates at the different quantile in model (16).

Control variables are introduced to optimize the model (15),

$$\begin{array}{c} \text{ln}\;Export_{it}=C+\beta_{1}\text{ln}\;gdp_{it}+\beta_{2}\text{ln}\;Chngdp_{t}+\gamma_{1}\text{ln}\;D_{i}+\gamma_{2}APEC_{i}+\gamma_{3}DC_{i}+\gamma_{4}\text{ln}\;Area_{i}+\alpha_{i}+\varepsilon_{it}, \end{array}$$ (16)

where *APEC* denotes whether the country/area is an APEC member, *DC* denotes whether the country/area is a developed country, and *Area* denotes the area of the country/area. The three newly added control variables are all time-invariant covariates. The F test and Hausman test results of (16) show that it should build the fixed effect panel model. The estimation results are plotted in Fig 2 using the W-QR estimation method proposed in this paper.

From Fig 2, we can see that the coefficient estimates of the distance variable are negative in the low and middle quartiles and become positive in the high quartiles. The trend of the coefficient estimates of the distance variable is similar to that in Fig 1, which indicates that the inhibitory effect of geographical distance is weakening for countries/regions with larger export trade. The estimated value of the coefficient of belonging to the APEC organization is positive at all quartiles, that is to say, belonging to the APEC organization has a boosting effect on international trade, indicating that the more developed the economy, the stronger the domestic consumption demand. The coefficient estimates of belonging to developed countries change from positive to negative as the quantile increases. The coefficient estimates of the area variable are negative at all quartiles, indicating that area has a depressing effect on China’s export trade, possibly because the larger a country is, the more abundant its production of materials and energy.

## Conclusion

In order to solve biased parameter estimation for time-invariant variables, this paper proposes two new panel quantile estimation methods, W-QR estimation method and W-IVQR estimation. The W-QR estimation method is applicable when the time-invariant variables are independent of fixed effects. W-IVQR estimation method is applicable to the case where the time-invariant variables are endogenous. The two new proposed methods combine the advantages of MD-QR estimation method and instrumental variable method, which can not only obtain effective estimation of time-varying covariate coefficients, but also be computationally convenient and simple to implement. In the first step, regress dependent variables on time-varying variables with an intercept using the conventional quantile regression to obtain the slope and intercept estimators for each individual. In the second step, use different weighted definitions to the obtained slope and intercept estimators to get the estimator of ***β*** and ***γ*** respectively. In the large sample property, the consistency of W-QR estimator and W-IVQR estimator under the sequential and simultaneous limits, and the asymptotic distribution of the two estimators under the simultaneous limit are studied. Monte Carlo simulation shows that W-QR estimator and W-IVQR estimator perform well in estimating coefficient of time-invariant variables. The W-QR and W-IVQR estimators for *β* and *γ* are asymptotically unbiased. At last, we illustrate the proposed W-QR estimation with an application to analyze the effects of the influence factors of China’s exports using the trade gravity model. We find that for countries/regions with large export trade volume, the inhibition of geographical distance is weakening, because the coefficient estimates of the distance variable are negative at the low and middle quartiles and become positive at the high quartiles.

## Appendix: Proofs

For convenience, we collect important definitions below. Let

$${\tilde{\text{z}}}_{i}={\text{z}}_{i}-\bar{\text{z}},\;{\tilde{\text{r}}}_{i}={\text{r}}_{i}-\bar{\text{r}},\;{\tilde{\eta }}_{i}\left(\tau \right)=\eta_{i}\left(\tau \right)-\bar{\eta }\left(\tau \right),\;\tilde{\hat{\alpha_{i}}}\left(\tau \right)=\hat{\alpha_{i}}\left(\tau \right)-\bar{\hat{\alpha }}\left(\tau \right),$$

$$\bar{\alpha }\left(\tau \right)=\sum_{i=1}^{N}\alpha_{i}\left(\tau \right),{\text{Q}}_{\text{r}\alpha (\tau )}=N^{-1}\sum_{i=1}^{N}({\text{r}}_{i}-\bar{\text{r}})(\alpha_{i}\left(\tau \right)-\bar{\alpha }\left(\tau \right){)}^{\prime },$$

$$\tilde{\gamma }\left(\tau \right)={\left({\text{Q}}_{\text{zr},N}{\text{Q}}_{\text{rr},N}^{-1}{\text{Q}}_{\text{zr},N}^{\prime }\right)}^{-1}\left({\text{Q}}_{\text{zr},N}{\text{Q}}_{\text{rr},N}^{-1}{\text{Q}}_{\text{r}\alpha (\tau ),N}\right).$$

**A.1. Consistency of** ${\hat{\gamma }}_{W-QR}\left(\tau \right)$ **and** ${\hat{\beta }}_{W-QR}\left(\tau \right)$ **under sequential asymptotics**

**Lemma 1**: As *N* → ∞,

$$\begin{array}{c} {\text{Q}}_{\text{zz},N}=\frac{1}{N}\sum_{i=1}^{N}{\tilde{\text{z}}}_{i}{\tilde{\text{z}}}_{i}^{\prime }\overset{p}{\to }{\text{Q}}_{\text{zz}}. \end{array}$$ (17)

**Proof**: We observe that $\frac{1}{N}\sum_{i=1}^{N}E\left[{\tilde{\text{z}}}_{i}{\tilde{\text{z}}}_{i}^{\prime }\right]\to {\text{Q}}_{\text{zz}}$ by Assumption 6(iii). Therefore, it is suffices to prove that

$$\begin{array}{c} \frac{1}{N}\sum_{i=1}^{N}\left({\tilde{\text{z}}}_{i}{\tilde{\text{z}}}_{i}^{\prime }-E\left[{\tilde{\text{z}}}_{i}{\tilde{\text{z}}}_{i}^{\prime }\right]\right)\overset{p}{\to }0. \end{array}$$ (18)

In turn, (18) follows from Assumption 6(ii) and 6(iii) and Chebyshev’s inequality. Hence, (18) follows. This completes the proof of the lemma.

Similarly, the matrix **Q**_**zr**,*N*_ and **Q**_**rr**,*N*_ are defined by

$${\text{Q}}_{\text{zr},N}=\frac{1}{N}\sum_{i=1}^{N}{\tilde{\text{z}}}_{i}{\tilde{\text{r}}}_{i}^{\prime },$$ (19)

$${\text{Q}}_{\text{rr},N}=\frac{1}{N}\sum_{i=1}^{N}{\tilde{\text{r}}}_{i}{\tilde{\text{r}}}_{i}^{\prime },$$ (20)

have finite probability limits as *N* → ∞ given by **Q**_**zr**_ and **Q**_**rr**_, that is ${\text{Q}}_{\text{zr},N}\overset{p}{\to }{\text{Q}}_{\text{zr}}$, ${\text{Q}}_{\text{rr},N}\overset{p}{\to }{\text{Q}}_{\text{rr}}$ where **Q**_**zr**_ and **Q**_**rr**_ appear in Assumption 6’.

**Lemma 2**: As *N* → ∞,

$$\frac{1}{N}\sum_{i=1}^{N}\eta_{i}(\tau )\eta_{i}(\tau ){\tilde{\text{z}}}_{i}{\tilde{\text{z}}}_{i}^{\prime }\overset{p}{\to }J\left(\tau \right).$$

**Proof**: Since

$$\frac{1}{N}\sum_{i=1}^{N}E\left[\eta_{i}(\tau )\eta_{i}(\tau ){\tilde{\text{z}}}_{i}{\tilde{\text{z}}}_{i}^{\prime }\right]\to J\left(\tau \right)$$

by Assumption 7(ii), it suffices to prove that

$$\begin{array}{c} \frac{1}{N}\sum_{i=1}^{N}(\eta_{i}(\tau )\eta_{i}(\tau ){\tilde{z}}_{i,k}{\tilde{z}}_{i,l}^{\prime }-E\left[\eta_{i}(\tau )\eta_{i}(\tau ){\tilde{z}}_{i,k}{\tilde{z}}_{i,l}^{\prime }\right])\overset{p}{\to }0 \end{array}$$ (21)

for *k*, *l* = 1, ⋯, *p* and given *τ* ∈ *U*.

As $E[{\left(\eta_{i}(\tau )\eta_{i}(\tau ){\tilde{z}}_{i,k}{\tilde{z}}_{i,l}^{\prime }\right)}^{2}]$ is not necessarily finite, let *δ* = *c*_*M*_/4. Then by H*ö*lder’s inequality,

$$\begin{array}{cc} & E[|\eta_{i}(\tau )\eta_{i}(\tau ){\tilde{z}}_{i,k}{\tilde{z}}_{i,l}{|}^{1+\delta }]\\ & \;\leq (E[|\eta_{i}(\tau ){|}^{4+4\delta }]\cdot E[|{\tilde{z}}_{i,k}{\tilde{z}}_{i,l}{|}^{2+2\delta }]{)}^{1/2}. \end{array}$$

In turn,

$$E[\underset{\tau \in U}{\text{sup}}|\eta_{i}\left(\tau \right){|}^{4+4\delta }]\leq C_{M},$$

$$E[|{\tilde{z}}_{i,k}{\tilde{z}}_{i,l}{|}^{2+2\delta }]\leq E[\|{\tilde{z}}_{i}{\|}^{4+4\delta }]\leq C_{M},$$

by Assumption 7(i) and 2(iv). Hence,

$$E[|\eta_{i}(\tau )\eta_{i}(\tau ){\tilde{z}}_{i,k}{\tilde{z}}_{i,l}{|}^{1+\delta }]\leq C_{M},$$

and so denoting $Z_{i}=\eta_{i}(\tau )\eta_{i}(\tau ){\tilde{z}}_{i,k}{\tilde{z}}_{i,l}-E[|\eta_{i}(\tau )\eta_{i}(\tau ){\tilde{z}}_{i,k}{\tilde{z}}_{i,l}|]$, we obtain

$$\begin{array}{c} E[|Z_{i}{|}^{1+\delta }]\leq C. \end{array}$$ (22)

With this notation, (21) is equivalent to $N^{-1}\sum_{i=1}^{N}Z_{i}\overset{p}{\to }0$.

Like Theorem 2.1.7 of Tao [27], for *G* > 0 to be chosen later, denote *Z*_*i*,≤*G*_ = *Z*_*i*_ ⋅ 1{|*Z*_*i*_| ≤ *G*} and *Z*_*i*,>*G*_ = *Z*_*i*_ ⋅ 1{|*Z*_*i*_| > *G*}. Then by Fubini’s theorem and Markov’s inequality,

$$\begin{array}{cc} \left|E[Z_{i,>G}]\right| & \leq E[|Z_{i,>G}|]\\ & =\int_{0}^{\infty }P(|Z_{i}|\cdot 1\{|Z_{i}|>G\}>s)ds\\ & =\int_{0}^{G}P(|Z_{i}|>G)ds+\int_{G}^{\infty }P(|Z_{i}|>s)ds\\ & =\int_{0}^{G}P(|Z_{i}^{1+\delta }|>G^{1+\delta })ds+\int_{G}^{\infty }P(|Z_{i}^{1+\delta }|>s^{1+\delta })ds\\ & \leq G\cdot \frac{E[|Z_{i}{|}^{1+\delta }]}{G^{1+\delta }}+\int_{G}^{\infty }\frac{E[|Z_{i}{|}^{1+\delta }]}{t^{1+\delta }}ds\\ & =\frac{E[|Z_{i}{|}^{1+\delta }]}{G^{\delta }}+\frac{E[|Z_{i}{|}^{1+\delta }]}{\delta G^{\delta }}\leq CG^{-\delta } \end{array}$$

where in the last inequality we used (22). Hence, by Markov’s inequality, for *ε* > 0,

$$P(|\frac{1}{N}\sum_{i=1}^{N}Z_{i,>G}|>\varepsilon )\leq \frac{1}{\varepsilon N}\sum_{i=1}^{N}E[|Z_{i,>G}|]\leq \frac{C}{\varepsilon G^{\delta }},$$

and since |*E*[*Z*_*i*,≤*G*_]| = |*E*[*Z*_*i*,>*G*_]| ≤ *CG*^−*δ*^,

$$\begin{array}{cc} P(|\frac{1}{N}\sum_{i=1}^{N}Z_{i,\leq G}|>\varepsilon +CG^{-\delta }) & \leq P(|\frac{1}{N}\sum_{i=1}^{N}(Z_{i,\leq G}-E[Z_{i,\leq G}])|>\varepsilon )\\ & \leq \frac{1}{\varepsilon^{2}N^{2}}\sum_{i=1}^{N}E\left[Z_{i,\leq G}^{2}\right]\\ & \leq \frac{G^{2}}{\varepsilon^{2}N^{2}}. \end{array}$$

Thus, setting *G* = *N*^1/3^, we obtain $N^{-1}\sum_{i=1}^{N}Z_{i}\overset{p}{\to }0$, which is is equivalent to (21).

**Proof of Theorem 1.1**. We first prove the consistency of ${\hat{\gamma }}_{W-QR}\left(\tau \right)$.

$$\begin{array}{cc} & {\hat{\gamma }}_{W-QR}\left(\tau \right)-\gamma \left(\tau \right)={\text{Q}}_{\text{zz},N}\sum_{i=1}^{N}{\tilde{\text{z}}}_{i}(\hat{\alpha_{i}}\left(\tau \right)-\bar{\hat{\alpha }}\left(\tau \right)-{\tilde{\text{z}}}_{i}^{\prime }\gamma \left(\tau \right))/N\\ & ={\text{Q}}_{\text{zz},N}\sum_{i=1}^{N}{\tilde{\text{z}}}_{i}(\hat{\alpha_{i}}\left(\tau \right)-{\text{z}}_{i}^{\prime }\gamma \left(\tau \right)-\left(\bar{\hat{\alpha }}\left(\tau \right)-{\bar{\text{z}}}^{\prime }\gamma \left(\tau \right)\right))/N\\ & ={\text{Q}}_{\text{zz},N}\sum_{i=1}^{N}{\tilde{\text{z}}}_{i}(\hat{\alpha_{i}}\left(\tau \right)-\alpha_{i}\left(\tau \right)+\eta_{i}\left(\tau \right)-\left(\bar{\hat{\alpha }}\left(\tau \right)-\bar{\alpha }\left(\tau \right)+\bar{\eta }\left(\tau \right)\right))/N\\ & ={\text{Q}}_{\text{zz},N}\sum_{i=1}^{N}{\tilde{\text{z}}}_{i}(\hat{\alpha_{i}}\left(\tau \right)-\alpha_{i}\left(\tau \right))/N-{\text{Q}}_{\text{zz},N}\sum_{i=1}^{N}{\tilde{\text{z}}}_{i}\left(\bar{\hat{\alpha }}\left(\tau \right)-\bar{\alpha }\left(\tau \right)\right)/N\\ & +{\text{Q}}_{\text{zz},N}\sum_{i=1}^{N}{\tilde{\text{z}}}_{i}\eta_{i}\left(\tau \right)/N-{\text{Q}}_{\text{zz},N}\sum_{i=1}^{N}{\tilde{\text{z}}}_{i}\bar{\eta }\left(\tau \right)/N\\ & ={\text{Q}}_{\text{zz},N}\sum_{i=1}^{N}{\tilde{\text{z}}}_{i}(\hat{\alpha_{i}}\left(\tau \right)-\alpha_{i}\left(\tau \right))/N+{\text{Q}}_{\text{zz},N}\sum_{i=1}^{N}{\tilde{\text{z}}}_{i}\eta_{i}\left(\tau \right)/N. \end{array}$$

The last equation holds because $\sum_{i=1}^{N}{\tilde{\text{z}}}_{i}=0$. Then, we show the first term is *o*_*p*_(1). By the consistency of quantile regression estimators, $(\hat{\alpha_{i}}\left(\tau \right)-\alpha_{i}\left(\tau \right))=o_{p}\left(1\right)$ as *T* → ∞. And $\|{\tilde{\text{z}}}_{i}\|\leq C_{M}$ by Assumption 6(ii), for fixed *N* as *T* → ∞, we have,

$${\text{Q}}_{\text{zz},N}\sum_{i=1}^{N}{\tilde{r}}_{i}(\hat{\alpha_{i}}\left(\tau \right)-\alpha_{i}\left(\tau \right))/N={\text{Q}}_{\text{zz},N}\sum_{i=1}^{N}o_{p}\left(1\right)/N.$$

Let *N* → ∞, we have ${\text{Q}}_{\text{zz},N}\overset{p}{\to }{\text{Q}}_{\text{zz}}$ by Lemma 1. Thus, ${\text{Q}}_{\text{zz},N}\sum_{i=1}^{N}{\tilde{r}}_{i}(\hat{\alpha_{i}}\left(\tau \right)-\alpha_{i}\left(\tau \right))/N=o_{p}\left(1\right)$.

Next, we show ${\text{Q}}_{\text{zz},N}\sum_{i=1}^{N}{\tilde{\text{z}}}_{i}\eta_{i}\left(\tau \right)/N=o_{p}\left(1\right)$. Use Assumptions 7(ii), by Lindeberg’s Central Limit Theorem and Cram$\overset{´}{e}$r-Wold device, we have

$$\frac{1}{\sqrt{N}}\sum_{i=1}^{N}{\tilde{\text{z}}}_{i}\eta_{i}\left(\tau \right)\overset{d}{\to }N\left(0,J\left(\tau \right)\right).$$

Let *N* → ∞, ${\text{Q}}_{\text{zz},N}\overset{p}{\to }{\text{Q}}_{\text{zz}}$, and

$${\text{Q}}_{\text{zz},N}\sum_{i=1}^{N}{\tilde{\text{z}}}_{i}\eta_{i}\left(\tau \right)/N=O_{p}\left(N^{-1/2}\right)=o_{p}\left(1\right).$$

Thus, as (*T*, *N*)_*seq*_ → ∞, we obtain

$$\left({\hat{\gamma }}_{W-QR}\left(\tau \right)-\gamma \left(\tau \right)\right)=o_{p}\left(1\right).$$

It follows that ${\hat{\gamma }}_{W-QR}\left(\tau \right)\overset{p}{\to }\gamma \left(\tau \right)$ as (*T*, *N*)_*seq*_ → ∞.

On the one hand, for fixed *N*, by Assumption 5, we have ${\hat{\text{V}}}_{i}\overset{p}{\to }{\text{V}}_{i}$. On the other hand, by the consistency of quantile regression estimators, we obtain $\hat{\beta_{i}}\left(\tau \right)\overset{p}{\to }\beta (\tau )$ as *T* → ∞. As (*T*, *N*)_*seq*_ → ∞,

$${\hat{\beta }}_{W-QR}\left(\tau \right)={\left(\sum_{i=1}^{N}{\hat{\text{V}}}_{i}^{-1}\right)}^{-1}\sum_{i=1}^{N}{\hat{\text{V}}}_{i}^{-1}{\hat{\beta }}_{i}\left(\tau \right)\overset{p}{\to }{\left(\sum_{i=1}^{N}{\text{V}}_{i}^{-1}\right)}^{-1}\sum_{i=1}^{N}{\text{V}}_{i}^{-1}\beta \left(\tau \right)=\beta \left(\tau \right).$$

This completes the proof.

**Remark** Strictly speaking, Assumption 5 is not really necessary; ${\hat{\text{V}}}_{i}$ can converge to anything because the rightmost equality would hold as long as ${\hat{\beta }}_{i}\left(\tau \right)$ is consistent as *T* → ∞.

**A.2. Consistency of** ${\hat{\gamma }}_{W-QR}\left(\tau \right)$ **and** ${\hat{\beta }}_{W-QR}\left(\tau \right)$ **under joint asymptotics**

**Lemma 3** Under Assumptions 1–4 and Assumption 5’, we have $\underset{1\leq i\leq N}{\text{max}}\|{\hat{\tilde{\beta }}}_{i}-{\tilde{\beta }}_{i}\|=O_{p}\left(\sqrt{\frac{\text{log}\;N}{T}}\right)$.

**Proof** Lemma 3 implies the Lemma 5 of Galvao and Wang [4]. We verify the conditions. Conditions A1-A5 of [4] are implied by Assumptions 1–4; Condition A6’ of [4] is implied by Assumption 5’. Therefore, the Lemma follows.

**Proof of Theorem 1.2**. By the proof of Theorem 1.1, we have

$$\begin{array}{c} {\hat{\gamma }}_{W-QR}\left(\tau \right)-\gamma \left(\tau \right)={\text{Q}}_{\text{zz},N}\sum_{i=1}^{N}{\tilde{\text{z}}}_{i}(\hat{\alpha_{i}}\left(\tau \right)-\alpha_{i}\left(\tau \right))/N+{\text{Q}}_{\text{zz},N}\sum_{i=1}^{N}{\tilde{\text{z}}}_{i}\eta_{i}\left(\tau \right)/N. \end{array}$$

By Assumption 6(ii), ${\tilde{\text{z}}}_{i}$ is bounded by *C*_*M*_. With using the Lemma 3, which implies that $\underset{1\leq i\leq N}{\text{max}}\|{\hat{\alpha }}_{i}-\alpha_{i}\|=O_{p}\left(\sqrt{\frac{\text{log}\;N}{T}}\right)$, we have

$$\begin{array}{cc} & {\tilde{\text{z}}}_{i}(\hat{\alpha_{i}}\left(\tau \right)-\alpha_{i}\left(\tau \right))\\ & \leq \|{\tilde{\text{z}}}_{i}\|\underset{1\leq i\leq N}{\text{max}}\|\hat{\alpha_{i}}\left(\tau \right)-\alpha_{i}\left(\tau \right)\|\\ & =O_{p}\left(\sqrt{\frac{\text{log}\;N}{T}}\right). \end{array}$$

Because ${\text{Q}}_{\text{zz},N}\overset{p}{\to }{\text{Q}}_{\text{zz}}$,

$$\begin{array}{cc} & {\text{Q}}_{\text{zz},N}\sum_{i=1}^{N}{\tilde{\text{z}}}_{i}(\hat{\alpha_{i}}\left(\tau \right)-\alpha_{i}\left(\tau \right))/N\\ & =O_{p}\left(\sqrt{\frac{\text{log}\;N}{T}}\right)\\ & =o_{p}\left(1\right). \end{array}$$

The last equation holds by the assumption of the relative rate of *N* and *T* in the theorem. Therefore, ${\text{Q}}_{\text{zz},N}\sum_{i=1}^{N}{\tilde{\text{z}}}_{i}(\hat{\alpha_{i}}\left(\tau \right)-\alpha_{i}\left(\tau \right))/N=o_{p}\left(1\right)$.

Besides, as *N* → ∞, ${\text{Q}}_{\text{zz},N}\overset{p}{\to }{\text{Q}}_{\text{zz}}$, and

$${\text{Q}}_{\text{zz},N}\sum_{i=1}^{N}{\tilde{\text{z}}}_{i}\eta_{i}\left(\tau \right)/N=O_{p}\left(N^{-1/2}\right)=o_{p}\left(1\right).$$

Therefore, we obtain

$${\hat{\gamma }}_{W-QR}\left(\tau \right)-\gamma \left(\tau \right)=o_{p}\left(1\right).$$

Moreover, by Lemma 3, we have

$$\begin{array}{cc} & {\hat{\beta }}_{W-QR}\left(\tau \right)-\beta \left(\tau \right)={\left(\sum_{i=1}^{N}{\hat{\text{V}}}_{i}^{-1}\right)}^{-1}\sum_{i=1}^{N}{\hat{\text{V}}}_{i}^{-1}\left({\hat{\beta }}_{i}\left(\tau \right)-\beta \left(\tau \right)\right)\\ & ={\left(\sum_{i=1}^{N}{\hat{\text{V}}}_{i}^{-1}\right)}^{-1}\sum_{i=1}^{N}{\hat{\text{V}}}_{i}^{-1}O_{p}\left(\sqrt{\frac{\text{log}\;N}{T}}\right)\\ & =O_{p}\left(\sqrt{\frac{\text{log}\;N}{T}}\right)\\ & =o_{p}\left(1\right). \end{array}$$

The last equation holds by the assumption of the relative rate of *N* and *T* in the theorem. This completes the proof.

**A.3. Asymptotic normality of** ${\hat{\gamma }}_{W-QR}\left(\tau \right)$ **and** ${\hat{\beta }}_{W-QR}\left(\tau \right)$ **under joint asymptotics**

**Proof of Theorem 2**. We only proof the asymptotic normality of ${\hat{\gamma }}_{W-QR}\left(\tau \right)$, the asymptotic normality of ${\hat{\beta }}_{W-QR}\left(\tau \right)$ has be proved in Theorem 3.2 of [4]. By above, we have

$$\begin{array}{c} \sqrt{N}\left({\hat{\gamma }}_{W-QR}\left(\tau \right)-\gamma \left(\tau \right)\right)={\text{Q}}_{\text{zz},N}\sum_{i=1}^{N}{\tilde{\text{z}}}_{i}(\hat{\alpha_{i}}\left(\tau \right)-\alpha_{i}\left(\tau \right))/\sqrt{N}+{\text{Q}}_{\text{zz},N}\sum_{i=1}^{N}{\tilde{\text{z}}}_{i}\eta_{i}\left(\tau \right)/\sqrt{N}. \end{array}$$

First, we show ${\text{Q}}_{\text{zz},N}\sum_{i=1}^{N}{\tilde{\text{z}}}_{i}(\hat{\alpha_{i}}\left(\tau \right)-\alpha_{i}\left(\tau \right))/\sqrt{N}=o_{p}\left(1\right)$. Because ${\text{Q}}_{\text{zz},N}\overset{p}{\to }{\text{Q}}_{\text{zz}}$ and ${\tilde{\text{z}}}_{i}$ is bounded by *C*_*M*_, by Lemma 3,

$$\begin{array}{cc} & {\text{Q}}_{\text{zz},N}\sum_{i=1}^{N}{\tilde{\text{z}}}_{i}(\hat{\alpha_{i}}\left(\tau \right)-\alpha_{i}\left(\tau \right))/\sqrt{N}\\ & =O_{p}\left(\sqrt{\frac{N\;\text{log}\;N}{T}}\right)\\ & =o_{p}\left(1\right). \end{array}$$

The last equation holds by the assumption of the relative rate of *N* and *T* in the theorem.

Next, use Assumption 7(ii), by Lindeberg’s Central Limit Theorem and Cram*é*r-Wold device, we have

$$\frac{1}{\sqrt{N}}\sum_{i=1}^{N}{\tilde{\text{z}}}_{i}\eta_{i}\left(\tau \right)\overset{d}{\to }N\left(0,J\left(\tau \right)\right).$$

Besides, ${\text{Q}}_{\text{zz},N}\overset{p}{\to }{\text{Q}}_{\text{zz}}$ as *N* → ∞. Thus, by Slutsky’s theorem, we obtain

$$\begin{array}{c} \sqrt{N}\left(\hat{\gamma }\left(\cdot \right)-\gamma \left(\cdot \right)\right)\overset{d}{\to }N\left(0,\Omega \left(\tau \right)\right), \end{array}$$ (23)

where $\Omega \left(\tau \right)={\text{Q}}_{\text{zz}}J\left(\tau \right){\text{Q}}_{\text{zz}}^{\prime }$, **Q**_**zz**_ is defined in Assumption 6(iii) and *J*(*τ*) is defined in Assumption 7(ii).

Considering the asymptotic normality of ${\hat{\beta }}_{W-QR}\left(\tau \right)$, one can be refer to Theorem 3.2 of Galvao and Wang [4]. We verify the conditions. Conditions A1-A5 of [4] are implied by Assumptions 1–4; Condition A6’ of [4] is implied by Assumption 5’. Therefore, the Theorem 2 follows.

**A.4. Consistency of** ${\hat{\gamma }}_{W-IVQR}\left(\tau \right)$ **and** ${\hat{\beta }}_{W-IVQR}\left(\tau \right)$ **under sequential asymptotics**

**Proof of Theorem 3.1**. The proof consists of two parts. First, we show that ${\hat{\gamma }}_{W-IVQR}\left(\tau \right)-\tilde{\gamma }\left(\tau \right)=o_{p}\left(1\right)$. Second, we show that $\tilde{\gamma }\left(\tau \right)-\gamma (\tau )=o_{p}\left(1\right)$.

Step 1:

$$\begin{array}{cc} & {\hat{\gamma }}_{W-IVQR}\left(\tau \right)-\tilde{\gamma }\left(\tau \right)\\ & ={\left({\text{Q}}_{\text{zr},N}{\text{Q}}_{\text{rr},N}^{-1}{\text{Q}}_{\text{zr},N}^{\prime }\right)}^{-1}{\text{Q}}_{\text{zr},N}{\text{Q}}_{\text{rr},N}^{-1}\sum_{i=1}^{N}{\tilde{\text{r}}}_{i}^{\prime }\left({\hat{\alpha }}_{i}\left(\tau \right)-\bar{\hat{\alpha }}\left(\tau \right)-\alpha_{i}\left(\tau \right)+\bar{\alpha }\left(\tau \right)\right)/N\\ & ={\left({\text{Q}}_{\text{zr},N}{\text{Q}}_{\text{rr},N}^{-1}{\text{Q}}_{\text{zr},N}^{\prime }\right)}^{-1}{\text{Q}}_{\text{zr},N}{\text{Q}}_{\text{rr},N}^{-1}\sum_{i=1}^{N}{\tilde{\text{r}}}_{i}^{\prime }\left({\hat{\alpha }}_{i}\left(\tau \right)-\alpha_{i}\left(\tau \right)\right)/N. \end{array}$$

The last equation holds because $\sum_{i=1}^{N}{\tilde{\text{r}}}_{i}=0$. By the consistency of quantile regression estimators, $(\hat{\alpha_{i}}\left(\tau \right)-\alpha_{i}\left(\tau \right))=o_{p}\left(1\right)$ as *T* → ∞ and $\|{\tilde{\text{r}}}_{i}\|\leq C_{M}$, for fixed *N* as *T* → ∞ we have ${\tilde{\text{r}}}_{i}(\hat{\alpha_{i}}\left(\tau \right)-\alpha_{i}\left(\tau \right))=o_{p}\left(1\right)$.

We note that by Lemma 1 as *N* → ∞,

$$\begin{array}{c} \hat{\text{S}}=({\text{Q}}_{\text{zr},N}{\text{Q}}_{\text{rr},N}^{-1}{\text{Q}}_{xw,N}^{\prime }{)}^{-1}{\text{Q}}_{\text{zr},N}{\text{Q}}_{\text{rr},N}^{-1}\overset{p}{\to }({\text{Q}}_{\text{zr}}{\text{Q}}_{\text{rr}}^{-1}{\text{Q}}_{\text{zr}}^{\prime }{)}^{-1}{\text{Q}}_{\text{zr}}{\text{Q}}_{\text{rr}}^{-1}=\text{S}. \end{array}$$ (24)

Thus,

$$\begin{array}{c} \hat{\text{S}}\sum_{i=1}^{N}{\tilde{\text{r}}}_{i}(\hat{\alpha_{i}}\left(\tau \right)-\alpha_{i}\left(\tau \right))/N=o_{p}\left(1\right). \end{array}$$

Step 2:

$$\begin{array}{cc} & \left(\tilde{\gamma }\left(\tau \right)-\gamma \left(\tau \right)\right)=\hat{\text{S}}\sum_{i=1}^{N}{\tilde{\text{r}}}_{i}(\alpha_{i}\left(\tau \right)-\bar{\alpha }\left(\tau \right)-{\tilde{\text{z}}}_{i}^{\prime }\gamma \left(\tau \right)/N)\\ & =\hat{\text{S}}\sum_{i=1}^{N}{\tilde{\text{r}}}_{i}(\alpha_{i}\left(\tau \right)-{\text{z}}_{i}^{\prime }\gamma \left(\tau \right)-\left(\bar{\alpha }\left(\tau \right)-{\bar{\text{z}}}^{\prime }\gamma \left(\tau \right)\right)/N)\\ & =\hat{\text{S}}\sum_{i=1}^{N}{\tilde{\text{r}}}_{i}(\alpha_{i}\left(\tau \right)-\alpha_{i}\left(\tau \right)+\eta_{i}\left(\tau \right)-\left(\bar{\alpha }\left(\tau \right)-\bar{\alpha }\left(\tau \right)+\bar{\eta }\left(\tau \right)\right)/N)\\ & =\hat{\text{S}}\sum_{i=1}^{N}{\tilde{\text{r}}}_{i}\eta_{i}\left(\tau \right)/N-\hat{\text{S}}\sum_{i=1}^{N}{\tilde{\text{r}}}_{i}\bar{\eta }\left(\tau \right)/N\\ & =\hat{\text{S}}\sum_{i=1}^{N}{\tilde{\text{r}}}_{i}\eta_{i}\left(\tau \right)/N. \end{array}$$

The last equation holds because $\sum_{i=1}^{N}{\tilde{\text{r}}}_{i}=0$. Use Assumption 6’(ii), by Lindeberg’s Central Limit Theorem and Cram$\overset{´}{e}$r-Wold device, we have

$$\frac{1}{\sqrt{N}}\sum_{i=1}^{N}{\tilde{\text{r}}}_{i}\eta_{i}\left(\tau \right)\overset{d}{\to }N\left(0,\tilde{J}\left(\tau \right)\right),$$

where $\tilde{J}\left(\tau \right)$ is defined in Assumption 6’(ii). Thus, we have

$$\tilde{\gamma }\left(\tau \right)-\gamma \left(\tau \right)=O_{p}\left(N^{-1/2}\right)=o_{p}\left(1\right).$$

as *N* → ∞. Combining Step 1 and Step 2, we get ${\hat{\gamma }}_{W-IVQR}\left(\tau \right)\overset{p}{\to }\gamma \left(\tau \right)$.

For fixed *N*, by Assumption 5, we have ${\hat{\text{V}}}_{i}\overset{p}{\to }{\text{V}}_{i}$. By the consistency of quantile regression estimators, we obtain $\hat{\beta_{i}}\left(\tau \right)\overset{p}{\to }\beta \left(\tau \right)$ as *T* → ∞. As (*T*, *N*)_*seq*_ → ∞,

$${\hat{\beta }}_{W-IVQR}\left(\tau \right)={\left(\sum_{i=1}^{N}{\hat{\text{V}}}_{i}^{-1}\right)}^{-1}\sum_{i=1}^{N}{\hat{\text{V}}}_{i}^{-1}{\hat{\beta }}_{i}\left(\tau \right)\overset{p}{\to }{\left(\sum_{i=1}^{N}{\text{V}}_{i}^{-1}\right)}^{-1}\sum_{i=1}^{N}{\text{V}}_{i}^{-1}\beta \left(\tau \right)=\beta \left(\tau \right).$$

This completes the proof.

**A.5. Consistency of** ${\hat{\gamma }}_{W-IVQR}\left(\tau \right)$ **and** ${\hat{\beta }}_{W-IVQR}\left(\tau \right)$ **under jiont asymptotics**

**Proof of Theorem 3.2** By the proof of Theorem 3.1, we have $\left(\tilde{\gamma }\left(\tau \right)-\gamma \left(\tau \right)\right)=o_{p}\left(1\right)$. Then, by Lemma 1, Lemma 3 and ${\tilde{r}}_{i}$ is bounded, we have

$$\begin{array}{cc} \left({\hat{\gamma }}_{W-IVQR}\left(\tau \right)-\tilde{\gamma }\left(\tau \right)\right)=\hat{\text{S}}\sum_{i=1}^{N}{\tilde{\text{r}}}_{i}^{\prime }\left({\hat{\alpha }}_{i}\left(\tau \right)-\alpha_{i}\left(\tau \right)\right)/N=O_{p}\left(\sqrt{\frac{\text{log}\;N}{T}}\right) & =o_{p}\left(1\right). \end{array}$$

using the condition that *T* is faster than *N*^2^ log *N* in the theorem. Thus, $\hat{\gamma }{}_{W-IVQR}\left(\tau \right)-\gamma \left(\tau \right)=o_{p}\left(1\right)$.

What’s more, by Lemma 3, we have

$$\begin{array}{cc} & {\hat{\beta }}_{W-IVQR}\left(\tau \right)-\beta \left(\tau \right)={\left(\sum_{i=1}^{N}{\hat{\text{V}}}_{i}^{-1}\right)}^{-1}\sum_{i=1}^{N}{\hat{\text{V}}}_{i}^{-1}\left({\hat{\beta }}_{i}\left(\tau \right)-\beta \left(\tau \right)\right)\\ & ={\left(\sum_{i=1}^{N}{\hat{\text{V}}}_{i}^{-1}\right)}^{-1}\sum_{i=1}^{N}{\hat{\text{V}}}_{i}^{-1}O_{p}\left(\sqrt{\frac{\text{log}\;N}{T}}\right)\\ & =O_{p}\left(\sqrt{\frac{\text{log}\;N}{T}}\right)\\ & =o_{p}\left(1\right). \end{array}$$

The last equation holds by the assumption of the relative rate of *N* and *T* in the theorem. This gives the asserted claim.

**A.6. Asymptotic normality of** ${\hat{\gamma }}_{W-IVQR}\left(\tau \right)$ **and** ${\hat{\beta }}_{W-IVQR}\left(\tau \right)$ **under jiont asymptotics**

**Proof of Theorem 4**. The proof of the asymptotic normality of ${\hat{\beta }}_{W-IVQR}\left(\tau \right)$ is the same as that of ${\hat{\beta }}_{W-QR}\left(\tau \right)$, please refer to Theorem 3.2 of Galvao and Wang [4].

Finally, by Slutsky’s theorem, for any *τ* ∈ *U*, we obtain

$$\sqrt{N}\left({\hat{\gamma }}_{W-IVQR}\left(\tau \right)-\gamma \left(\tau \right)\right)\overset{d}{\to }N\left(0,\tilde{\Omega }\left(\tau \right)\right),$$

where $\tilde{\Omega }\left(\tau \right)=\text{S}\tilde{J}\left(\tau \right)\text{S}$, $\tilde{J}\left(\tau \right)$ is defined in Assumption 7’(ii) and $\text{S}=({\text{Q}}_{\text{zr}}{\text{Q}}_{\text{rr}}^{-1}{\text{Q}}_{\text{zr}}^{\prime }{)}^{-1}{\text{Q}}_{\text{zr}}{\text{Q}}_{\text{rr}}^{-1}$.

## Supporting information

S1 Dataset Export data dataset. (XLS)
